# Diversity of cultivable fungal endophytes in *Paullinia cupana* (Mart.) Ducke and bioactivity of their secondary metabolites

**DOI:** 10.1371/journal.pone.0195874

**Published:** 2018-04-12

**Authors:** Fábio de Azevedo Silva, Rhavena Graziela Liotti, Ana Paula de Araújo Boleti, Érica de Melo Reis, Marilene Borges Silva Passos, Edson Lucas dos Santos, Olivia Moreira Sampaio, Ana Helena Januário, Carmen Lucia Bassi Branco, Gilvan Ferreira da Silva, Elisabeth Aparecida Furtado de Mendonça, Marcos Antônio Soares

**Affiliations:** 1 Departamento de Botânica e Ecologia, Instituto de Biociências, Universidade Federal de Mato Grosso, Cuiabá, Mato Grosso, Brasil; 2 Instituto Federal de Mato Grosso, Cáceres, Mato Grosso, Brasil; 3 Escola de Ciências Biológicas e Ambientais, Universidade Federal de Grande Dourados, Dourados, Mato Grosso do Sul, Brasil; 4 Faculdade de Medicina, Universidade Federal de Mato Grosso, Cuiabá, Mato Grosso, Brasil; 5 Departamento de Química, Universidade Federal de Mato Grosso, Cuiabá, Mato Grosso, Brasil; 6 Centro de Pesquisa em Ciências Exatas e Tecnológicas, Universidade de Franca, Franca, São Paulo, Brasil; 7 Embrapa Amazônia Ocidental, Manaus, Amazonas, Brasil; 8 Faculdade de Agronomia, Universidade Federal de Mato Grosso, Cuiabá, Mato Grosso, Brasil; Universita degli Studi di Pisa, ITALY

## Abstract

*Paullinia cupana* is associated with a diverse community of pathogenic and endophytic microorganisms. We isolated and identified endophytic fungal communities from the roots and seeds of *P*. *cupana* genotypes susceptible and tolerant to anthracnose that grow in two sites of the Brazilian Amazonia forest. We assessed the antibacterial, antitumor and genotoxic activity *in vitro* of compounds isolated from the strains *Trichoderma asperellum* (1BDA) and *Diaporthe phaseolorum* (8S). In concert, we identified eight fungal species not previously reported as endophytes; some fungal species capable of inhibiting pathogen growth; and the production of antibiotics and compounds with bacteriostatic activity against *Pseudomonas aeruginosa* in both susceptible and multiresistant host strains. The plant genotype, geographic location and specially the organ influenced the composition of *P*. *cupana* endophytic fungal community. Together, our findings identify important functional roles of endophytic species found within the microbiome of *P*. *cupana*. This hypothesis requires experimental validation to propose management of this microbiome with the objective of promoting plant growth and protection.

## Introduction

*Paullinia cupana* var. *sorbilis (Mart*.*)* Ducke is a Brazilian Amazonia plant species. Commonly known as guaraná, it is used as central nervous system stimulant due to its high concentration of caffeine [1]. The first *P*. *cupana* crops were grown by indigenous tribes, but the beverage industry has prompted the development of commercial-scale crops. Nowadays, guaraná seeds not only represent a global trend in the market of soft and energy drinks [2], but are also promising raw materials for the development of drugs and supplements with different properties, such as anti-fatigue [3,4], antioxidant [5–7], antimicrobial [8–10], antiproliferative [11], anxiolytic [12] and cardioprotective [13,14]. However, plant diseases like anthracnose and oversprouting, caused by *Colletotrichum guaranicola* F. C. Albuq and *Fusarium decemcellulare* Brick, respectively, have seriously damaged *P*. *cupana* crops and limited their expansion and productivity [15–18]. There are promising alternatives for disease control–such as cultivation of resistant *P*. *cupana* genotypes [19,20]–or the biological control of phytopathogens by endophytic microrganisms [21,22].

Endophytes are microorganisms that inhabit the inner tissue of their hosts, at least during a phase of the endophyte’s life cycle, and perform various ecological relationships without showing visible symptoms of infection in the host [23–25]. Endophytes are intensively studied for bioprospecting purposes, but their interaction with the host and associated ecological factors in tropical regions remain poorly investigated [26–28]. Some studies have reported the endophytic fungal diversity of *P*. *cupana* leaves [18,29,30] and endophytic bacterial diversity of *P*. *cupana* leaves, seeds and roots [31,32]. However, many aspects of the *P*. *cupana* endophytic fungal community have not yet been assessed, such as: (i) whether abiotic factors, such as weather, geographic distance, plant tissue, ultraviolet radiation and culture system influence its endophytic fungal community’s structure and composition, as observed in other host plants [33–38]; (ii) whether different *P*. *cupana* genotypes host different endophytic fungal communities, as occurs in other crops [39,40]; and (iii) the bioprospecting potential and functional traits of *P*. *cupana* endophytes [30,32,41].

Several functional traits such as synthesis of hydrolytic enzymes and indole acetic acid are related to the protection against phytopathogens, plant growth promotion and resistance to environmental stress, which are relevant for their multiple applications in agriculture [42–44]. The same is true for natural products synthesized by endophytes, which have important applications in human health [45–50]. There is a strong demand for new and more effective antibiotics and anticancer drugs, which can be synthesized by endophytes [51–54].

Considering that different factors can influence the structure of endophytic communities, the present study aims to identify cultivable endophytic fungal species in *P*. *cupana* roots and seeds and to examine how the plant organ, genotype and geographic location influence the endophytic fungal community structure and production of functional traits for plant growth promotion and induction of tolerance to pathogens. In addition, we have isolated and identified the major special metabolites from two endophytic fungal species (*Trichoderma asperellum* and *Diaporthe phaseolorum*) and examined their antibacterial, antitumor and genotoxic activity *in vitro*.

## Materials and methods

### Isolation and molecular identification of endophytic fungi from *P*. *cupana* roots and seeds

This study was carried out with genotypes obtained by vegetative propagation (clonal cultivar) to ensure the analysis of the same genetic material in plants grown in two different places. The seeds and roots of five adult healthy plants of *P*. *cupana* from each clonal cultivars CMU 871 (tolerant to anthracnose and oversprouting) and CMU 300 (tolerant to anthracnose and susceptible to oversprouting) [16,55] were collected in November 2014 in the Embrapa (Brazilian Agricultural Research Company) experimental farms located in the cities of Manaus (2° 53' 29.14''S and 59° 58' 39.90''W, 99 m high) and Maués (3° 22' 54'' S and 57° 42' 55'' W, 18 m high), State of Amazonas, Brazil. The plant material was collected at the end of the dry season, when the accumulated rainfall and average temperature were respectively 196.0 mm and 28.46°C in Manaus, and 272.3 mm and 28.17°C in Maués [56,57].

The plant material was stored and transported under refrigeration until processing. Roots and seeds were washed in tap water and neutral detergent. Surface disinfection was performed in a laminar flow hood by soaking in 70% ethanol for 30 s and 2.5% NaClO for 8 min (roots) or 20 min (seeds), and rinsing five times with sterile distilled water [58]. The efficacy of surface disinfection was verified by inoculating the last rinse water on tryptone soy agar (TSA) plates. Disinfected seeds and roots were inoculated on potato dextrose agar (PDA) and TSA culture medium (Kasvi, Curitiba, PR, Brazil) supplemented with streptomycin, chloramphenicol and tetracycline (200 mg/L), and incubated for 15 days. The fungal strains were grouped morphologically [59], and the grouping was confirmed by analyzing microscopic traits in microculture slides [60] and comparing the obtained results with taxonomic keys [61,62]. The isolates were stored under refrigeration in PDA medium and in tubes with sterilized rice. DNA of one strain from each morphological group was extracted using the Genomic DNA Isolation Kit (Norgen Biotek Corporation, Thorold, ON, Canada), according to the manufacturer’s protocol. The internal transcribed spacer regions (ITS) were amplified using the ITS1 and ITS4 primers [63,64]. The polymerase chain reaction products were purified using ExoSAP-IT (USB Corp., Cleveland, OH, USA) and sequenced by the Sanger method, using the Big Dye Terminator kit (Applied Biosystems, Foster City CA, USA). The obtained sequences were analyzed with BioEdit Sequence Alignment Editor (version 7.2.5) and compared with sequences deposited in the NCBI GenBank database (National Center for Biotechnology Information).

### Community structure analysis

The endophytic colonization rate [65] and the relative frequency of fungal species [28] were evaluated. The species diversity, richness and dominance were estimated using the non-parametric Shannon-Weaver index [66], Chao 1 index [67] and Simpson index [68], respectively. The degree of similarity among different communities was determined using the Jaccard index [69], while the UPGMA (Unweighted Pair Group Method with Arithmetic Mean) cluster analysis grouped the endophytes into distinct clusters. To examine whether a given endophytic community plays predominant roles, we analyzed their ecological roles. The species were classified into the categories phytopathogen, saprophyte, mycotroph, entomopathogen and coprophilous, according to the criteria established by literature reports and the Agricultural Research Service of the United States Department of Agriculture (USDA-ARS) database [70].

Non-metric multidimensional scaling (NMDS) based on Jaccard's coefficient was used to visually detect the preference of the species in relation to each host plant geographic location, genotype and organ. The distinctiveness of fungal assemblages was tested with one-way analysis of similarity (ANOSIM) considering the incorporation of 999 permutations. The indicator value method (IndVal) [71] was used to identify the most characteristic species of each community, and the ones more prevalent in a single community. Permutation test was performed to confirm whether the indicator value was statistically significant (*p* < 0.05).

A network was built to analyze the existence of interspecific interactions. Correlation between species were considered positive when the Spearman correlation coefficient (ρ) was > 0.6, and statistically significant when *p* < 0.05. The rate of connectivity between nodes was determined by analyzing betweenness centrality, while the Louvain algorithm was used to detect modularity [72].

### Root associations, endophytic potential and functional traits

*P*. *cupana* roots were washed, clarified with KOH (10%, v/v), and stained with trypan blue in lactoglycerol (0.05%, v/v) for visualization of fungal-root associations [73]. Fifteen root fragments from each individual were analyzed in the Nikon Eclipse E200 microscope coupled with a camera (Bel Photonics), in order to identify the presence of dark septate endophytic fungi (DSE) and endomycorrhizal structures, such as arbuscles and vesicles. The identity of the structures was confirmed by comparison with literature reports [74].

Endophytic fungi with dark mycelium and septate hyphae isolated from *P*. *cupana* were selected to examine their infection capacity and identify typical structures of dark septate endophytes. *P*. *cupana* seeds were not used due to the difficulty in promoting their germination [75], and alternatively, seeds of the *Sorghum* cultivar BRS 373 were used (*Sorghum* sp.). *Sorghum* sp seeds were disinfected [76] and incubated in minimum mineral medium (MM) [77] at room temperature (approximately 27°C) under natural light. After 5 days, the rootlets of seedlings were inoculated with mycelium fragments. Non-inoculated seedlings were used as controls. The roots were cleared, stained, and analyzed after 15 days of interaction [73]. Stained roots were preserved in polyvinyl lactoglycerol and observed under light microscope [78].

The production of cellulase [79], esterase [80], amylase, phosphatase and protease [81] was analyzed *in vitro* in all the isolated endophytic fungal species, using essentially qualitative tests. The siderophore production was determined in cromoazurol S medium [82]. Discs of endophytic mycelium (⌀1 mm) were used as inoculum and the formation of halos was determined after 24 h incubation. The production of indoleacetic acid (IAA) was determined by a colorimetric method, using BD broth supplemented with tryptophan (0.73 μmol/ml) as the negative control [83]. The IAA concentration was determined using a standard curve prepared with commercial IAA.

### Antibacterial activity

A lineage of each endophytic fungal species was grown in PDA medium for the production of ethyl acetate (EtOAc) extracts, as reported by Rosa [84]. The qualitative antibacterial activity of the endophytes’ EtOAc extracts (20 mg/mL) was assessed as reported by Ichikawa [85], against the following strains: susceptible *Escherichia coli* (ATCC 25922), *Pseudomonas aeruginosa* (ATCC 9027) and *Staphylococcus aureus* (ATCC 6538), and multiresistant *E*. *coli* (1A), *P*. *aeruginosa* (2A) and *S*. *aureus* (3A). The extracts that suppressed bacterial growth the most strongly were selected for determination of the minimal inhibitory concentration (MIC)–the lowest sample concentration at which bacteria do not grow [86]–using serial dilutions ranging from 5,000 μg/mL to 39 μg/mL. Tetracycline (0.05 mg/mL) and LB broth were used as the positive and negative controls, respectively. The minimum concentration of death (MCD) was determined using Mueller-Hinton agar medium [87].

### Purification and structural elucidation of special metabolites

The species *Trichoderma asperellum* (1BDA) and *Diaporthe phaseolorum* (8S) were selected for the chemical analysis because they exhibited significant functional traits. Crude EtOAc extracts from *D*. *phaseolorum* (8S) (235 mg) and *T*. *asperellum* (1BDA) (240 mg) were prepared as reported by Rosa [84] and further purified through reversed-phase column chromatography using C-18 resin (230–400 mesh, Merck) as stationary phase and CH_3_OH:H_2_O (3:7, 7:3 and 10:0 v/v) as eluent.

High-performance liquid chromatography (HPLC) was performed in the Varian ProStar 215 chromatograph and data were acquired using the software Galaxie Chromatography Data System (Varian). The CH_3_OH fraction from *T*. *asperellum* (1BDA) was subjected to semi-preparative HPLC (Phenomenex column, 10.0 × 250 mm, 5 μm, C18, equipped with a precolumn) under elution with CH_3_OH/H_2_O/HCO_2_H (7:2,9:0.1) in isocratic condition to afford 1-hydroxy-8-methoxyanthraquinone (sample 17A, 1.5 mg) as a light-yellow solid.

The CH_3_OH:H_2_O fractions from *D*. *phaseolorum* (8S) were submitted to column chromatography employing silica gel 60 (40–63 mesh, Merck). The fraction CH_3_OH:H_2_O (7:3) was eluted with EtOAc:CH_3_OH (1:1 v/v), hexane and CH_3_OH to provide the di-(2-ethylhexyl)phthalate (DEHP, sample 070, 2.1 mg) as a dark yellow solid. The fraction CH_3_OH:H_2_O (3:7) was sequentially eluted with hexane:EtOAc (6:4) and CHCl_3_ to furnish 3-hydroxypropionic acid (3HPA, sample 3A, 2.7 mg) as a colorless solid.

Nuclear magnetic resonance (NMR) spectra were recorded using a Bruker Ascend 500 spectrometer operating at 500 MHz for ^1^H nuclei and 125 MHz for ^13^C nuclei. Chemical shifts were quoted in parts per million (ppm), referenced to the appropriate residual solvent peak. Two-dimensional spectroscopy (COSY, HSQC and HMBC) were used for structural determination. Gas chromatography coupled to mass spectrometry (GC-MS) were performed on a Shimadzu GC17A chromatograph coupled to a GCMSQP5050A detector. After structural determination, the purified samples were tested for antibacterial activity using the method reported in the previous section. MIC was determined with serial dilutions ranging from 30 μg/mL to 0.2 μg/mL.

### Antitumor activity

The elucidated purified molecules (DEHP and 3HPA) were tested for antitumor activity. The Chinese hamster ovary cells (CHO) and *Mus musculus* skin melanoma B16F10 cell lines were kindly provided by Dr. Edgar Julian Paredes Gamero from Federal University of São Paulo (UNIFESP, São Paulo, SP, Brazil) and cultured in the Laboratory of Cell Culture and Molecular Biology at the Federal University of Grande Dourados (Dourados, MS, Brazil). CHO and B16F10 cells were cultured in DMEM + F10 and RPMI-1640 medium, respectively, containing 10% fetal bovine serum (FBS), 100 U/mL penicillin and 100 μg/mL streptomycin, in a humidified incubator at 37°C and under 5% CO_2_.

The 3-(4,5-dimethyl-2-thiazolyl)-2,5-diphenyl-2*H*-tetrazolium bromide (MTT) assay provides a sensitive measurement of the cell metabolic status, in particular the mitochondrial function, which reflects early cellular redox changes [88]. CHO and B16F10 cells (6x10^3^ and 1x10^3^cells/well, respectively) were grown in 96-well tissue culture plates and treated with the isolated compounds (3.12, 6.25, 12.5, 25, 50 and 100 μg/mL) for 24, 48 and 72 h. The cells were washed with 0.1 mL of 0.1 M phosphate buffered saline (PBS), pH 7.4, at 37°C, and further incubated with 100 μL of MTT solution (1 mg/mL in culture medium) for 3 h, at 37°C. The assay medium was removed and the dark-blue formazan crystals formed in intact cells were dissolved in DMSO. The absorbance was recorded at 630 nm in a microplate reader. Three independent experiments were performed in triplicate. Doxorubicin was used as positive control; it inhibits CHO and B16F10 cell growth by 50% (IC_50_) at the concentration of 1 and 0.1 μg/mL, respectively. The results were expressed as the percentage of MTT reduction relative to the control group (untreated cells).

### Genotoxicity

The alkaline comet assay [89] was used to assess the genotoxicity of compound 3A isolated from *D*. *phaseolorum* (8S) EtOAc extract. This is a sensitive method capable of detecting DNA strand breaks, alkali-labile sites and DNA-DNA/DNA-protein cross-linking [90]. A prerequisite to perform this assay is cell viability >70% because cytotoxic concentrations of a given sample may cause DNA damage that does not reflect the genotoxic effect. Thus, first we used the MTT assay to address whether compound 3A was cytotoxic towards V79 cells. Briefly, after a 24-h treatment with this sample (12.5, 25.0, 50.0 or 100.0 μg/mL) at 36.5°C, the assay medium was removed and the cells were incubated with 100 μL of MTT (0.5 mg/mL) for 2 h at 36.5°C. The assay medium was replaced by 100 μL of DMSO and, after five minutes, the samples were transferred to another plate to record their absorbance at 540 nm, using a microplate reader (Multiskan EX, Thermo Electron Corporation). Untreated cells and 10.0 μM doxorubicin (Sigma-Aldrich, St. Louis, MO, USA) represented the negative and positive controls, respectively. The results were expressed as the percentage of MTT reduction relative to the negative control group (100% survival).

The comet assay was performed using the method reported by Speit [91], with minor modifications. The V79 cells were treated with sample 3A (25.0, 50.0, or 100.0 μg/mL) for 3 h, at 36.5°C, protected from light to prevent additional DNA damage. Cells treated with medium and 200 μM H_2_O_2_ were used as the negative and positive controls, respectively. Aliquots of 20 μL of cell suspension were mixed with 100 μL of low-melting point agarose (0.5% in PBS) and layered onto a microscope slide previously thin-layered with 1.5% normal-melting point agarose. The slides were immersed in freshly prepared lysing solution (100 mM EDTA, 10 mM Tris, 2.5 M NaCl, 1% Triton X-100, and 10% DMSO pH 10 –all from Sigma-Aldrich, St. Louis, MO, USA) for at least 24 h, at 4°C. Then, the slides were immersed in alkaline buffer (0.2 M EDTA, 10 M NaOH, pH >13, both from Sigma-Aldrich, St. Louis, MO, USA) for 25 min, at 4°C, and submitted to electrophoresis at 0.92 V/cm and 300 mA, for further 25 min. Next, the slides were immersed in neutralization buffer (0.4 M Tris-HCl, pH 7.5, Sigma-Aldrich, St. Louis, MO, USA) for 15 min, and fixed in 100% ethanol for 5 min.

The slides were air-dried, stained with 0.3X Sybr Gold Nucleic Acid Gel Stain (Invitrogen, Carlsbad, CA), and analysed in an Ecliple Ci fluorescence microscope (Nikon, Japan) equipped with a 20X objective. Images of at least 100 nucleoids were obtained using the Lucia Comet Assay software (version 7.30, Laboratory Imaging, Czech Republic). The % DNA in the comet tail was considered as the parameter of genotoxicity. Three independent experiments were assayed in triplicate. Once the negative control of one experiment was excluded due to technical problems, we carried out an additional experiment without replicates. The data from each experiment are presented as the mean of median of 100 or 200 nucleoids.

### Statistical analysis

The Past software [92] was used to determine diversity and equitability indexes and perform cluster analysis. The package vegan in the R software version 3.3.2 [93] was used to carry out the analyses of variance, similarity, permutation and correlation. Indicator species test was performed using package labdsv (http://ecology.msu.montana.edu/labdsv/R) within the R environment. Network was constructed and visualized using the software Gephi version 0.9.1 [94]. Venn diagrams were generated with DrawVenn web tool (http://bioinformatics.psb.ugent.be/webtools/Venn/). Data from the antitumor activity assay were analyzed by the Dunnett's multiple comparison test, using the GraphPad Prism software (GraphPad Software, Inc., San Diego, CA). Data from the comet assay (both cytotoxicity and genotoxicity) presented normal distribution and were statisticaly compared using one-way ANOVA and Bonferroni’s post-hoc test, at a significance level of 5%, with the aid of the GraphPad Prism software.

## Results and discussion

### Diversity of endophytic fungi in *P*. *cupana* roots and seeds

We isolated and identified 256 strains and 34 species of endophytic fungi from *P*. *cupana* roots and seeds. The phylum Ascomycota, the class Sordariomycetes, and the order Diaporthales had the highest relative frequency (Table 1). The aforementioned taxa often colonize tropical plant communities [28,95].

**Table 1:** Endophytic fungal taxa isolated with the highest relative frequency (%) from *Paullinia cupana*, stratified according to the plant organ, genotype and geographic location.

Taxa	Organ		Genotype		Location	
	Roots	Seeds	Susceptible	Tolerant	Manaus	Maués
Phylum	Ascomycota (99.5)	Ascomycota (93.7)	Ascomycota (98.4)	Ascomycota (99.1)	Ascomycota (99.2)	Ascomycota (98.3)
Class	Sordariomycetes (59.6)	Sordariomycetes (87.5)	Sordariomycetes (67.4)	Sordariomycetes (56.9)	Sordariomycetes (62.9)	Sordariomycetes (60.4)
Order	Diaporthales (25.1)	Diaporthales (53.1)	Diaporthales (27.2)	Diaporthales (30)	Diaporthales (26.7)	Diaporthales (29.8)
Family	Diaporthaceae (20.6)	Diaporthaceae (53.1)	Diaporthaceae (21.2)	Diaporthaceae (28.4)	Diaporthaceae (20.4)	Diaporthaceae (29.8)
Genus	*Xylogone* (34.9)	*Diaporthe* (40.6)	*Xylogone* (25.7)	*Diaporthe* (14.6)	*Xylogone* (29.1)	*Xylogone* (30)
Species	*Xylogone ganodermophthora* (34.9)	*Diaporthe phaseolorum* (34.3)	*Xylogone ganodermophthora* (25.7)	*Xylogone ganodermophthora* (35.7)	*Xylogone ganodermophthora* (29.13)	*Xylogone ganodermophthora* (33)

The genus *Xylogone* and the species *Xylogone ganodermophthora* had the highest total relative frequency in root endophytic communities (Table 1). The genus *Xylogone* was described only once as endophyte of *Taxus chinensis* [96], and *X*. *ganodermophthora* was described as mycopathogen [97] and as an antifungal species against watermelon pathogens [98]. Here we report for the first time that the species *X*. *ganodermophtora* is an endophyte of *P*. *cupana*. *Phomopsis asparagi*, the species with the second highest total relative frequency (Table 2), was reported as endophyte [99] and phytopathogen in asparagus crops [100].

**Table 2:** Relative frequency (%) of endophytic fungal species isolated from different microbial communities in *Paullinia cupana*.

Species–Strain	Manaus				Maués				Total Frequency	GenBank Accession number
	Susceptible		Tolerant		Susceptible		Tolerant			
	Root	Seed	Root	Seed	Root	Seed	Seed	Root		
***Xylogone ganodermophthora*– 64BDA**	22.2	0	46.9	0	38.5	0	35	0	30.8	KU512708
***Phomopsis asparagi*– 34TSA**	9.5	20	4.1	0	15.4	20	20	0	12.5	KU512684
***Fusarium oxysporum*– 20S**	11.1	0	10.2	0	9.6	10	8.3	0	9	KU512674
***Periconia macrospinosa*– 66BDA**	7.9	0	2	0	5.8	0	10	0	5.9	KU512687
***Diaporthe phaseolorum*– 8S**	0	20	0	66.7	0	10	0	25	4.3	KU512679
***Mariannaea camptospora*– 123BDA**	7.9	0	2	0	3.8	0	1.7	0	3.5	KU512698
***Diaporthe hongkongensis*– 11BDA**	1.6	0	4.1	0	3.8	0	6.7	0	3.5	KU512691
***Trichoderma harzianum*– 43BDA**	4.8	0	0	0	3.8	0	3.3	0	2.7	KU512711
***Humicola fuscoatra*– 124BDA**	7.9	0	0	0	3.8	0	0	0	2.7	KU512712
***Mycoleptodiscus terrestris* - 64TSA**	7.9	0	2	0	0	0	1.7	0	2.7	KU512714
***Diaporthe melonis* - 29TSA**	1.6	0	2	0	1.9	0	3.3	0	2	KU512682
***Sydowiella fenestrans* - 104BDA**	6.3	0	2	0	0	0	0	0	2	KU512692
***Melanconiella elegans* - 113BDA**	6.3	0	2	0	0	0	0	0	2	KU512673
***Phomopsis lagerstroemiae* - 43TSA**	0	0	0	0	0	0	6.7	0	1.6	KU512690
***Glomerella acutata* - 18TSA**	0	20	4.1	0	1.9	0	0	0	1.6	KU512686
***Gibberella zeae* - 3S**	0	0	0	8.3	0	0	0	50	1.2	KU512707
***Paraphaeosphaeria arecacearum* - 45TSA**	0	0	2	0	3.8	0	0	0	1.2	KU512678
***Nigrograna mackinnonii* - 101BDA**	0	0	4.1	0	0	0	1.7	0	1.2	KU512680
***Pestalotiopsis microspora*– 14S**	0	0	0	16.7	0	0	0	25	1,2	KU512681
***Arxiella dolichandrae* - 90BDA**	1.6	0	4.1	0	0	0	0	0	1.2	KU512704
***Trichoderma asperellum* - 1BDA**	0	0	0	0	3.8	0	0	0	0.8	KU512700
***Fusarium polyphialidicum* - 5S**	0	20	0	8.3	0	0	0	0	0.8	KU512705
***Diaporthe terebinthifolii* - 24S**	0	20	0	0	0	10	0	0	0.8	KU512670
***Fusarium solani* - 41BDA**	1.6	0	2	0	0	0	0	0	0.8	KU512702
***Peyronellaea pinodella* - 28S**	0	0	0	0	0	20	0	0	0.8	KU512671
***Fomitopsis meliae* - 32S**	0	0	0	0	0	20	0	0	0.8	KU512693
***Parapleurotheciopsis inaequiseptata* - 62TSA**	0	0	2	0	0	0	0	0	0.4	KU512695
***Colletotrichum gloeosporioides* - 6TSA**	0	0	2	0	0	0	0	0	0.4	KU512696
***Mycena robusta* - 95BDA**	0	0	2	0	0	0	0	0	0.4	KU512710
***Pochonia boninensis* - 49BDA**	0	0	0	0	0	0	1.7	0	0.4	KU512701
***Paecilomyces parvisporus* - 9BDA**	0	0	0	0	1.9	0	0	0	0.4	KU512703
***Nectria rigidiuscula* - 29S**	0	0	0	0	0	10	0	0	0.4	KU512689
***Penicillium janthinellum* - 17BDA**	1.6	0	0	0	0	0	0	0	0.4	KU512715
***Talaromyces pinophilus* - 12TSA**	0	0	0	0	1.9	0	0	0	0.4	MF167569

*Diaporthe phaseolorum* and *P*. *asparagi* were the species with the highest relative frequency in seed endophytic communities (Table 2). Only the former was present in all the endophytic communities of *P*. *cupana* seeds (Table 2). The genus *Phomopsis* and the species *D*. *phaseolorum* are predominantly endophytic in many tropical species [101–104].

Only the genera *Diaporthe*, *Fusarium*, *Glomerella* and *Phomopsis* were identified in endophytic communities of both the roots and seeds of *P*. *cupana* (Table 2). The genera *Diaporthe*, *Pestalotiopsis* and *Phomopsis* were previously identified in endophytic communities of phyllospheres [29]. Thus, we hypothesize that endophytic fungi that colonize *P*. *cupana* have organ-specificity, as reported for other tropical and temperate host plants [105–107].

Most of the endophytic species occurred at a total relative frequency lower than 10%. The species *Arxiella dolichandrae*, *Mariannaea camptospora*, *Mycena robusta*, *Paraphaeosphaeria arecacearum*, *Parapleurotheciopsis inaequiseptata*, *Peyronellaea pinodella* and *Pochonia boninensis* occur in tropical regions [108–114], while *X*. *ganodermophthora* occurs only in temperate regions [115]. Nevertheless, their occurrence in endophytic communities has not been reported yet, indicating that *P*. *cupana* is a repository of fungal species in the Brazilian Amazonia forest. As *Peyronellaea pinodella* [116] and *Arxiella dolichandrae* [117] have already been reported as phytopathogens in other hosts, we believe that they are endophytes or latent pathogens in *P*. *cupana* because the plant-endophyte relationship can vary from mutualism to parasitism [42,118]; however, this hypothesis requires experimental validation.

Regarding the ecological role of the isolated species (S1 Table), all the endophytic fungal communities isolated from *P*. *cupana* had at least one species that is reported as endophyte, phytopathogen, saprophyte, mycoparasite, entomopathogen or coprophile. Some fungal species have been reported as both endophytic and phytopathogenic. It is known that endophytic communities present latent phytopathogens [119] and several phytopathogens can behave as endophytes depending on the local biotic and abiotic conditions [120,121]. In addition, the genetic diversity of some endophytic fungi may enable them to act as phytopathogens or saprophytes [122], and some phytopathogens may not be able to develop the disease due to host defense mechanisms [123,124].

Several species reported as saprophytes, coprophiles and entomopathogens were isolated from *P*. *cupana* (S1 Table). Coprophilous fungi are transmitted to plant tissues by herbivores [125], while saprophytic fungi mainly derive from soil and rhizosphere [126,127]. Entomopathogenic fungi may have an endophytic phase of life [128] and some endophytic fungal genera have entomopathogenic species [129]. Endophytes with entomopathogenic potential are frequently tested for their ability to inhibit agricultural pests [127,130,131]. These reports indicate that *P*. *cupana* may host endophytic fungal species with potential for pest control; this hypothesis requires further experimental validation.

Mycoparasitic species may be abundant in endophytic communities [132–134], such as *Colletotrichum gloeosporioides* [135], *Fusarium oxysporum* [136], *Humicola fuscoatra* [137], *Talaromyces pinophilus* [138], *Trichoderma asperellum* [139], *Trichoderma harzianum* [140], and *Xylogone ganodermophthora* [115]. Several species of mycoparasitic fungi behave as endophytes, such as *C*. *gloeosporioides* [141], *H*. *fuscoatra* [142], *T*. *asperellum* [143], and *T*. *harzianum* [144].

Analysis of the indicator value revealed that few species preferred a specific community. Regarding the geographic location, *P*. *asparagi* significantly preferred Maués (IndVal = 0.314, *P* = 0.01). Regarding the *P*. *cupana* organs, *X*. *ganodermophthora* significantly preferred root communities (IndVal = 0.475, *P* = 0.001), while *D*. *phaseolorum* significantly preferred seed communities (IndVal = 0.15, *P* = 0.001). *H*. *fuscoatra* was significantly associated with the root endophytic communities of the susceptible *P*. *cupana* genotype grown in Manaus (IndVal = 0.2, *P* = 0.012). No species was significantly associated with a specific genotype (susceptible or tolerant).

The *P*. *cupana* genotype influenced the endophytic fungal community diversity (Table 3). Considering the communities from seeds collected in both geographic locations (Manaus and Maués), those isolated from the susceptible genotype exhibited higher richness, Shannon index and evenness when compared with those isolated from the tolerant genotype. Regarding the root endophytic communities from Manaus, those isolated from the susceptible *P*. *cupana* genotype had higher Shannon index, evenness and colonization rate than those isolated from the tolerant genotype. The endophytic communities isolated from the roots and seeds of the susceptible *P*. *cupana* genotype have higher richness and Shannon index than those isolated from the same organs of the tolerant genotype. The richness of microbial communities in the tolerant genotype was greater than that of the susceptible genotype only in the root endophytic community of plants grown in Manaus. The root endophytic communities isolated from the susceptible genotype grown in Maués had higher richness, Shannon index and dominance but lower evenness and colonization rate than the root endophytic communities from the tolerant genotype grown in the same location.

**Table 3:** Alpha diversity and colonization rate (%) of endophytic fungal communities isolated from *P*. *cupana*.

Diversity index	Manaus				Maués			
	Susceptible		Tolerant		Susceptible		Tolerant	
	Root	Seed	Root	Seed	Root	Seed	Root	Seed
**Total species**	15	5	18	4	13	7	12	3
**Total strains**	63	5	49	12	51	10	60	4
**Shannon index**	2.43	1.6	2.11	0.98	2.04	1.88	1.98	1.04
**Chao index**	25	15	27.1	4.5	13.4	8.5	14	3.5
**Simpson index**	0.89	0.8	0.75	0.51	0.79	0.84	0.8	0.62
**Colonization rate**	24.6	1.9	19.1	4.6	19.9	3.9	23.4	1.5

**Fig 1:**
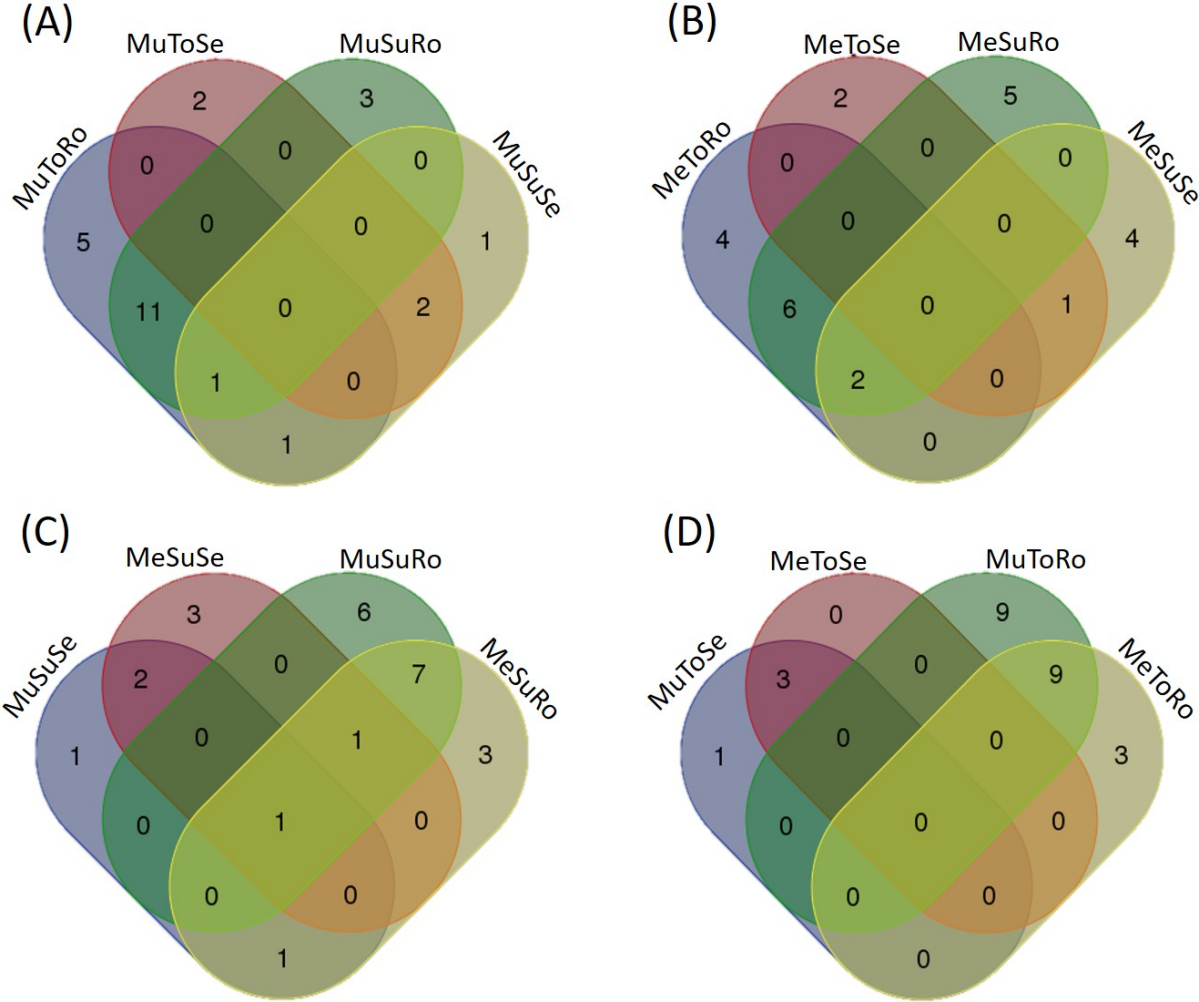
Venn diagrams representing the distribution of endophytic fungal species in the communities studied. (A) Susceptible and tolerant genotypes from Manaus, (B) Susceptible and tolerant genotypes from Maués, (C) Roots and seeds from susceptible genotypes, (D) Roots and seeds from tolerant genotypes. Mu = Manaus; Me = Maués; Su = Susceptible genotype (CMU 300); To = Tolerant genotype (CMU 871); Se = seed; Ro = Root.

The root endophytic communities of the tolerant and susceptible genotypes grown in Manaus shared 11 species (Fig 1A), while those grown in Maués shared 6 species (Fig 1B). Communities isolated from the same organ shared a higher number of species in the susceptible (Fig 1C) and tolerant (Fig 1D) genotypes. Only the species *F*. *oxysporum*, *G*. *acutata* and *P*. *asparagi* occurred in both organs, explaining why communities of different organs shared few species. The root endophytic communities of plants grown in Manaus had the largest number of unique species (Fig 1C and 1D).

As reported in tobacco, tomato and potato cultivars [39,40,145], we found that different endophytic communities colonized the susceptible and tolerant genotypes of *P*. *cupana*, and that the susceptible genotypes exhibited higher species diversity–the last finding may be related to their vulnerability to microbial infections, which colonize tissues faster [146,147]. Moreover, roots can produce exudates that promote colonization of certain groups of microorganisms [148,149]. Exudates produced by different genotypes can influence the microbial community in rhizosphere and consequently affect the composition and richness of endophytic communities [150]. Additional studies are required to determine whether susceptible and tolerant *P*. *cupana* genotypes differ with respect to the composition of exudates.

The geographic location where *P*. *cupana* was grown also influenced the endophytic fungal community’s diversity (Table 3). Compared with the same genotype grown in Maués, the endophytic fungal communities isolated: (i) from seeds of the tolerant genotype grown in Manaus had higher colonization rate, dominance and richness; (ii) from seeds of the susceptible genotype grown in Manaus had lower colonization rate, evenness, richness and Shannon index; (iii) from roots of the tolerant genotype grown in Manaus had higher dominance, richness and Shannon index; (iv) from roots of the susceptible genotype grown in Manaus had higher colonization rate, evenness, richness and Shannon index.

The observed and estimated species richness (Chao index) in the endophytic fungal communities identified in Maués were similar to each other; in Manaus, the estimated richness was higher than that observed for most endophytic communities (Table 3). Therefore, the geographic location influences the endophytic fungal diversity in *P*. *cupana*. Manaus exhibited the highest species richness, more specifically in seeds of the tolerant genotype and roots of the susceptible genotype.

The level of particulate matter in the air of urban areas is high due to several anthropic activities, such as industrial and vehicular emissions, biomass burning and soil resuspension [151,152]. Particulate matter suspended in the air carries a high diversity of fungi and bacteria [153–156] that can colonize the surface and integrate the epiphytic community of plants on which the particles deposit [157]. Several studies have reported epiphytic species as endophytes [28,158,159], indicating that a variety of species can colonize the inner plant tissues. Considering that Maués is located in a rural area within the Amazonia forest relatively distant from urban centers, and that Manaus is a large urban center, we hypothesized that the *P*. *cupana* samples grown in Manaus received more particulate matter from the air and thereby a greater amount of inoculum, which may explain their greater richness of endophytic fungal species. Urban and rural areas may present distinct endophytic communities even among hosts belonging to the same species [160–162].

Other factors such as temperature, rainfall and soil composition can influence the diversity of endophytic species [163–165]. These parameters differed between Manaus and Maués, which may have impacted on the endophytic diversity of each community. According to Koppen climate classification, Manaus climate is ranked as Am (monsoon), with average annual temperature of 26.7°C, average annual rainfall of 2.420 mm and soil characterized as yellow latosol. On the other hand, Maués climate is ranked as Af (humid equatorial), with average annual temperature of 25.5°C, mean annual rainfall of 2.101 mm and soil characterized as eutrophic gleysoil [166,167].

The plant organ is an important determining factor of the endophytic community’s composition in *P*. *cupana*, because the roots were colonized by communities with higher richness and diversity (S1 Fig). The isolation of seeds from the external environment for a long period [168] and the presence of antifungal compounds in seeds [9] may also lower endophytic species richness in this plant organ. *P*. *cupana* seeds are surrounded by a thick epicarp and partially surrounded by a thin membrane (the pith) [75], while the roots are in constant contact with the ground and can be infected by a great diversity of edaphic microorganisms.

Although the NMDS plot did not show separation between the endophytic fungal communities of distinct *P*. *cupana* genotypes and geographic locations, the communities of seeds and roots were clearly different and clustered as distinct groups (ANOSIM R^2^ = 0.27, *P* = 0.001, stress = 0.064) (S2 Fig). The root endophytic communities had a narrower distribution because they shared greater similarity in composition and abundance, indicating that the plant organ influences the community’s composition in *P*. *cupana*.

**Fig 2:**
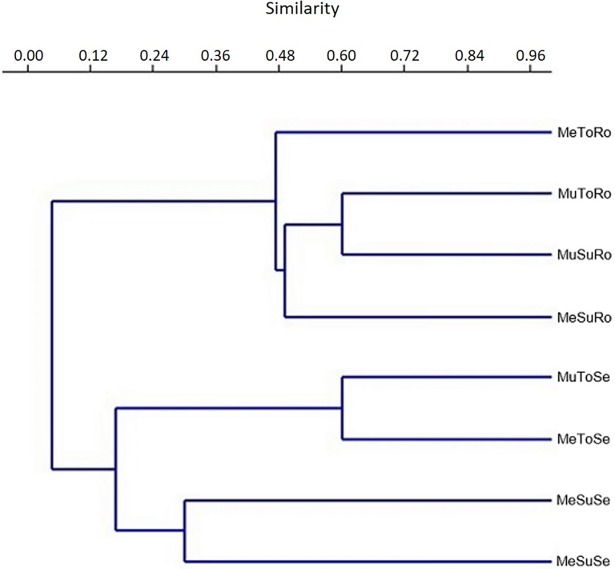
Analysis of the similarity degree between endophytic communities isolated from the seeds and roots of two *P*. *cupana* genotypes grown in Manaus and Maués. Mu = Manaus; Me = Maués; Su = Susceptible phenotype (CMU 300); To = Tolerant phenotype (CMU 871); Se = Seeds; Ro = Roots.

The Jaccard index of similarity (Fig 2) showed that (i) the communities from seeds and roots shared the lowest levels of similarity; (ii) the clonal type was crucial to distinguish the communities within each plant organ; and (iii) the location determined the highest rates of similarity between communities. The degree of similarity between endophytic communities may decrease as the geographical distance between same host species increases [28,34,35,165,169].

Regarding the network analysis, the 34 nodes were connected by 107 edges, with average degree of 6.29 and clustering coefficient of 0.617 (S3 Fig). Ten species presented intermediate values of betweenness centrality, most of them were isolated from *P*. *cupana* roots and occured in both clonal types grown in both locations. *X*. *ganodermophtora* and *P*. *asparagi* were also identified in both clonal types from both locations and exhibited the highest betweenness centrality. *X*. *ganodermophtora* had the highest centrality degree, with 23 edges, binding to almost all species isolated from roots and acting as a local hub, while *P*. *asparagi* had the second highest centrality degree, with 20 edges mainly composed of species isolated from seeds. The network had two modules: the largest and the smallest ones were composed of endophytic fungal species isolated from *P*. *cupana* roots and seeds, respectively (S4 Fig). *P*. *asparagi* constituted an important node because it connected the seed and the root species modules. The formation of distinct modules for each organ, as well as the presence of different indicator species in each organ, stressed that different communities colonize the roots and seeds. Therefore, *X*. *ganodermophtora* and *P*. *asparagi* represented the central species of the endophytic fungal community of *P*. *cupana* and exerted the strongest influence on its structure, stability and dynamics.

### *In vitro* endophytic ability

**Fig 3:**
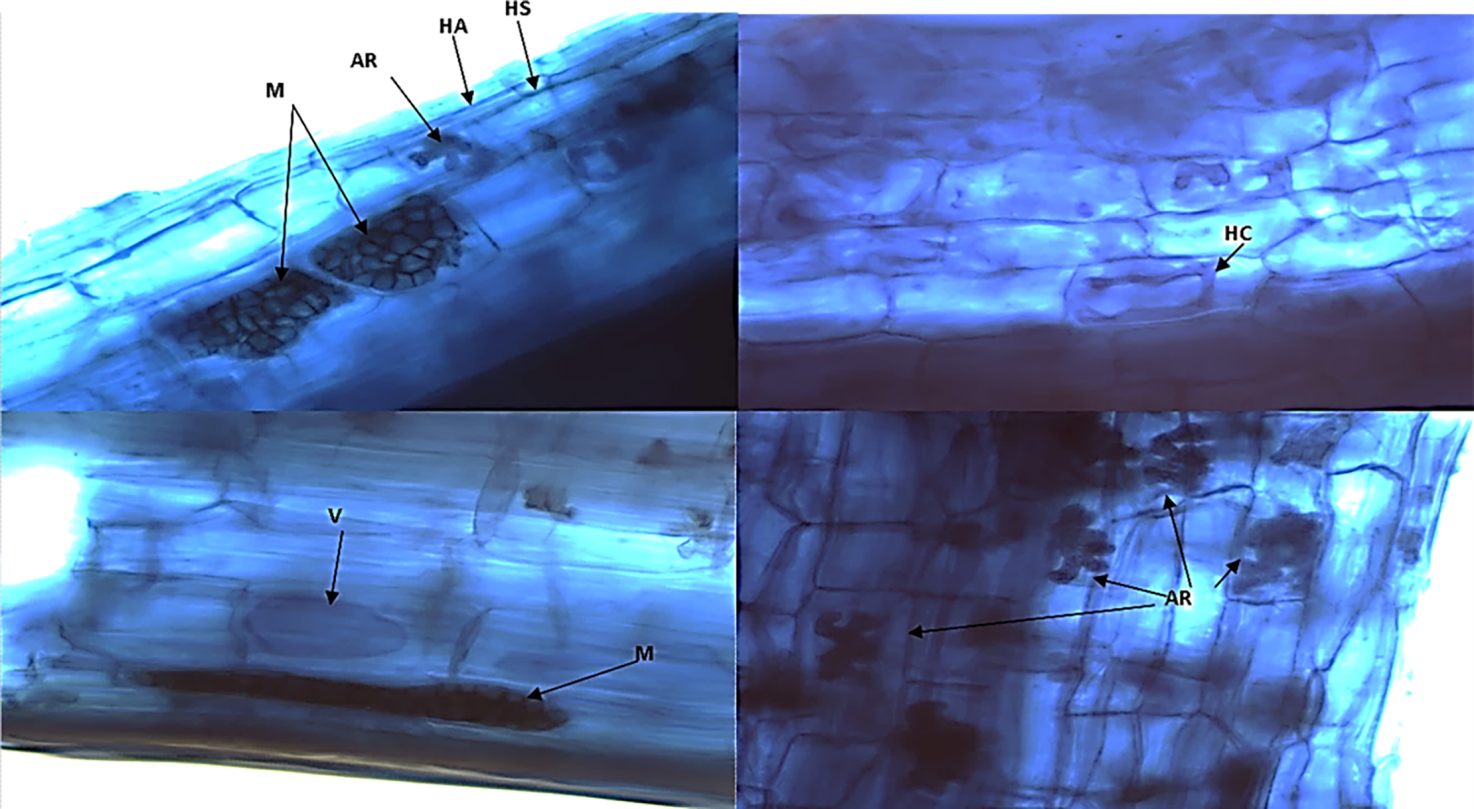
Structure of mycorrhizal fungi and dark septate endophytic fungi detected within *P*. *cupana* roots (40x). Arbuscle (AR), Coenocytic hyphae (HA), Coil hyphae (HC), Microsclerotium (M), Septate brown hyphae (HS), Vesicle (V).

The presence of microsclerotia in the roots of all samples analyzed confirmed that *P*. *cupana* was associated with DSE (Fig 3). Associations with DSE fungi can become pathogenic, although most of them benefit the host by increasing nutrients absorption and promoting plant growth [170–172].

*P*. *cupana* roots contained typical structures of mycorrhizal fungi. Arbuscules with the *arum* type morphology were detected in the roots of the susceptible genotype grown in Manaus; coil hyphae and brown septate hyphae were exclusively detected in tolerant host plants; and vesicles were observed only in the clonal cultivar grown in Maués. The literature reports the association between *P*. *cupana* and mycorrhizal fungi [173,174]. Colonization and sporulation are seasonal events strongly influenced by increased rainfall [173], favored by increased soil acidity and manganese concentration, and inhibited by high iron content [174].

**Fig 4:**
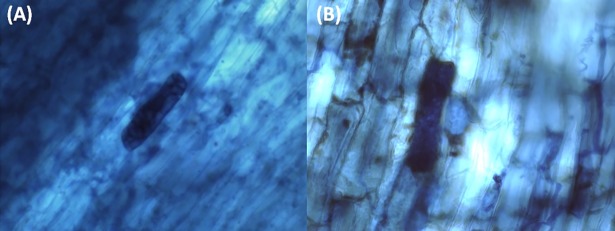
Microsclerotium in sorghum roots (40x). (A) *Phomopsis asparagi* (22TSA) and (B) *Peyronellaea pinodella* (28S).

The strains *C*. *gloeosporioides* (6TSA), *P*. *asparagi* (22TSA) *P*. *pinodella* (28S), and S*ydowiella fenestrans* (104BDA) were selected for the evaluation of endophytic capacity because they presented dark mycelium and septate hyphae. *P*. *asparagi* is frequently reported as phytopathogenic against asparagus crops [100], causing discoloration of the stem or trunk, oval lesions and brown spots on the roots [175,176]. Although the endophyte *P*. *asparagi* (22TSA) produced microsclerotia (Fig 4A), it also caused the same pathogenic symptoms reported for asparagus, probably due to the long cultivation period used in this study.

Sorghum roots colonized by *P*. *pinodella* (28S) contained microsclerotia (Fig 4B), but they did not show apparent symptoms of disease. Among the endophytic fungal species isolated from seeds, only *P*. *pinodella* (28S) exhibited dark mycelium and septate hyphae, the other fungal species colonized the seedling radicle without either developing dark septate structures or causing disease symptoms. There are no literature reports that the fungal species evaluated produce DSE structures; however, there are DSE species belonging to the genus *Diaporthales* [177], *Phomopsis* [178] and *Pleosporales* [179,180].

DSE are distributed in a wide variety of environments, such as sub-Antarctic regions, temperate and tropical forests, semi-arid regions, among others [181,182]. The currently available information on DSE mainly comes from studies carried out in arctic and temperate regions [183]. Several tropical plant families associate with DSE [181], but there are few reports on the association between DSE and species of the family *Sapindaceae* [184,185] and other species in the Amazonia region [186,187].

### Analysis of functional traits

The EtOAc extract of the 27 endophytic fungal species isolated from *P*. *cupana* inhibited bacterial growth in the qualitative test (Table 4), and the EtOAc extract of 37 fungal strains suppressed the growth of at least one bacterial strain. Only *D*. *phaseolorum* (8S) inhibited the growth of *P*. *aeruginosa* susceptible and multiresistant strains. The antibacterial activity of several endophytic fungal species that we isolated was previously reported in the literature, such as *C*. *gloeosporioides* [188], *D*. *melonis* [189], *D*. *phaseolorum* [103], *F*. *meliae* [190], *F*. *oxysporum* [191], *F*. *polyphialidicum* [192], *F*. *solani* [193], *G*. *acutata* [194], *G*. *zeae* [195], *H*. *fuscoatra* [196], *M*. *camptospora* [197], *P*. *janthinellum* [198], *P*. *microspora* [199], *T*. *asperellum* [29] and *T*. *harzianum* [200]. To the best of our knowledge, this is the first report on the antibacterial activity of *A*. *dolichandrae*, *D*. *hongkongensis*, *D*. *terebinthifolii*, *M*. *terrestris*, *M*. *robusta*, *N*. *mackinnonii*, *P*. *asparagi*, *P*. *arecacearum*, *P*. *boninensis*, *P*. *inaequiseptata*, *P*. *parvisporus* and *X*. *ganodermophthora*. Only the antifungal activity of *X*. *ganodermophthora* was reported previously [201].

**Table 4:** Analysis of functional traits in endophytic fungi isolated from *Paullinia cupana*.

Species–Strain	Antibacterial activity (MIC in μg/mL)^a^				Enzymatic activity^b^			
	E.C (M)	E.C (S)	S.A (M)	S.A (S)	Amylase	Cellulase	Protease	IAA^c^
*Phomopsis asparagi* - 22S	-	-	-	+	-	+	+	+
*Diaporthe phaseolorum -* 8S	39.06	> 5.000	1.250	1.250	-	-	+	+
*Mycoleptodiscus terrestris* - 65TSA	+	+	-	+	-	-	-	-
*Pestalotiopsis microspore* - 14S	-	-	-	+	+	+	+	+
*Glomerella acutata -* 18TSA	+	+	-	+	-	-	-	-
*Colletotrichum gloeosporioides* - 6TSA	-	+	-	-	-	-	-	-
*Gibberella zeae* - 3S	-	-	-	+	-	-	+	+
*Nigrograna mackinnonii* - 101BDA	-	-	-	+	-	-	-	+
*Fomitopsis meliae* - 32S	-	-	-	+	-	+	-	-
*Phomopsis asparagi -* 38TSA	-	-	-	+	-	-	-	-
*Glomerella acutata* - 98BDA	-	-	-	+	-	-	-	-
*Xylogone ganodermophthora* - 92BDA	-	-	-	+	-	-	-	-
*Parapleurotheciopsis inaequiseptata* - 62TSA	-	-	-	+	-	-	+	-
*Diaporthe melonis* - 29TSA	312.5	39.06	> 5.000	5.000	+	-	+	+
*Paraphaeosphaeria arecacearum* - 45TSA	-	-	-	+	-	-	-	-
*Mycoleptodiscus terrestris* - 30TSA	-	-	-	+	+	+	+	+
*Paecilomyces parvisporus* - 9BDA	-	-	-	+	-	+	-	-
*Arxiella dolichandrae* - 90BDA	-	-	-	+	-	+	+	-
*Phomopsis asparagi -* 34TSA	-	+	-	+	-	-	-	-
*Pochonia boninensis* - 49BDA	-	-	-	+	-	-	+	-
*Fusarium solani* - 41BDA	-	-	-	+	+	+	-	+
*Mycena robusta* - 95BDA	-	-	-	+	+	-	-	+
*Diaporthe hongkongensis* - 11BDA	-	-	-	+	-	-	+	+
*Mariannaea camptospora* - 14TSA	-	-	-	+	-	-	-	+
*Fusarium oxysporum* - 20S	-	-	-	+	+	+	+	+
*Fusarium polyphialidicum* - 5S	-	-	-	+	-	+	+	-
*Penicillium janthinellum* - 17BDA	-	-	-	+	+	+	-	-
*Trichoderma harzianum* - 43BDA	78.13	> 5.000	2.500	5.000	+	+	+	+
*Humicola fuscoatra* - 124BDA	-	-	-	+	-	-	+	+
*Diaporthe terebinthifolii -* 24S	78.13	2.500	> 5.000	625	-	-	-	-
*Trichoderma asperellum* - 1BDA	-	-	-	+	-	-	-	+
*Glomerella acutata* - 15S	-	-	-	+	-	+	+	+
*Mariannaea camptospora* - 123BDA	-	-	+	+	-	-	-	+
*Fusarium oxysporum* - 13TSA	-	-	-	+	-	-	-	+
*Xylogone ganodermophthora -* 1TSA	-	-	+	+	-	-	-	-
*Xylogone ganodermophthora* - 130BDA	-	-	+	+	-	-	-	-
*Xylogone ganodermophthora* - 11TSA	-	-	-	+	-	+	+	-

^a^ (+) = Inhibit gowth

(-) = Do not inhibit growth; E.C = *Escherichia coli*; S.A = *Staphylococcus aureus*; (M) = Multiresistant strain; (S) = Susceptible strain; (MIC) = Minimal inhibitory concentration.

^b^ (+) = Produce the enzyme; (-) = Do not produce the enzyme.

^c^ IAA = indole-3-acetic acid.

The EtOAc extracts of *D*. *melonis* (29TSA), *D*. *phaseolorum* (8S), *D*. *terebinthifolii* (24S), and *T*. *harzianum* (43BDA)–which suppressed the growth of the greatest number of bacterial strains in the qualitative test–were selected for determination of the minimal inhibitory concentration (Table 4). Analysis of the minimum concentration of death evidenced some microbial growth after treatment with all the extracts’ concentrations tested, indicating that they exerted bacteriostatic activity and that their minimum concentration of death was higher than 5.000 μg/mL (Table 4). The antimicrobial activity of *D*. *melonis* [189], *D*. *phaseolorum* [103] and *T*. *harzianum* [202–204] has already been reported.

We identified 8 amylase-, 13 cellulase-, and 15 protease-producing endophytic fungal species in *P*. *cupana* (Table 4). Here we report for the first time the production of (i) amylase by *D*. *melonis*, *M*. *robusta* and *M*. *terrestris*; (ii) cellulase by *A*. *dolichandrae*, *F*. *polyphialidicum*, *P*. *parvisporus*, *P*. *microspora*, *P*. *asparagi* and *X*. *ganodermophthora*; (iii) and protease by *A*. *dolichandrae*, *D*. *hongkongensis*, *D*. *melonis*, *D*. *phaseolorum*, *F*. *polyphialidicum*, *H*. *fuscoatra*, *M*. *terrestris*, *P*. *asparagi*, *P*. *boninensis*, *P*. *inaequiseptata*, *P*. *microspora* and *X*. *ganodermophthora*. Some of the species that we identified are known for the production of (i) amylase, such as *F*. *oxysporum*, *F*. *meliae*, *F*. *solani*, *G*. *zeae*, *P*. *janthinellum* and *T*. *harzianum* [205–209]; (ii) protease, such as *F*. *meliae*, *F*. *oxysporum*, *F*. *solani*, *G*. *acutata*, *G*. *zeae*, *M*. *camptospora* and *P*. *janthinellum*, *T*. *harzianum* [205,210–214]; and (iii) cellulase, such as *D*. *phaseolorum*, *F*. *meliae*, *F*. *oxysporum*, *F*. *solani*, *G*. *acutata*, *G*. *zeae*, *H*. *fuscoatra*, *M*. *terrestris* and *T*. *harzianum* [205,215–222].

We did not identify esterase-, phosphatase-, and siderophore-producing endophytic fungal species in *P*. *cupana* under the conditions assessed. Thirteen endophytic fungal species did not display any enzyme activity; however, enzyme release by some of them were already reported, such as the synthesis of (i) amylase and cellulase by *C*. *gloeosporioides* and *P*. *macrospinosa* [205,223]; (ii) amylase and protease by *M*. *camptospora* [214,224]; and (iii) amylase, cellulase and protease by *T*. *asperellum* [225–227]. The species *D*. *terebinthifolii*, *M*. *elegans*, *N*. *rigidiuscula*, *N*. *mackinnonii*, *P*. *arecacearum*, *P*. *macrospinosa*, *P*. *pinodella*, *P*. *lagerstroemiae* and *S*. *fenestrans*–which did not present enzymatic activity in our work–, are not reported in the literature with any enzymatic activity.

Only the species *F*. *oxysporum*, *M*. *terrestris* and *T*. *harzianum*, which were isolated from tolerant and susceptible genotypes of *P*. *cupana*, exhibited amylolytic, cellulolytic and proteolytic activity. Although protease-, cellulase-, and amylase-producing endophytic fungal species colonized both *P*. *cupana* genotypes, the production of enzymes and antifungals in the roots may not be sufficient to prevent the development of anthracnose–this disease affects the aerial organs, mostly leaflets, petioles and young stems [18,30,228].

We identified 17 IAA-producing endophytic fungal species in *P*. *cupana* (Table 4), among which *F*. *oxysporum*, *F*. *solani*, *T*. *harzianum* and *T*. *asperellum* are known IAA producers *in vitro* [229–231]. Although the other endophytic fungal species analyzed did not produce detectable levels of IAA, several species of *Glomerella*, *Mycena* and *Pestalotiopsis* were previously reported to synthesise IAA *in vitro* and promote plant growth [232–234]. Endophytes can release IAA to promote plant growth in several crops such as rice, sugar cane [235] and coffee [236], and to favor germination [237]. Together, our findings demonstrate that endophytic fungi isolated from tolerant and susceptible genotypes of *P*. *cupana* also synthesize IAA *in vitro*, which could benefit the host.

### Purification and structural elucidation of special metabolites

The CH_3_OH-H_2_O (3:7) fraction from *D*. *phaseolorum* (8S) afforded a colorless solid, named sample 3A. The ^1^H NMR spectrum showed two signals at 3.07 ppm (2H, t, J = 6.02 Hz) and 4.68 ppm (2H, t, J = 6.05 Hz); the ^13^C NMR spectrum showed three signals at 30.74, 69.29 and 174.51 ppm; and the 2D NMR spectrum showed two triplets coupled with each other (COSY). HMQC correlations demonstrated that protons with chemical shifts at 4.68 and 3.07 ppm were attached to the carbons that gave the signals at 69.29 and 30.74 ppm, respectively, while the HMBC correlation demonstrated that both proton signals gave long-range correlations to each other’s carbons as well as to the carbon at 174.51 ppm. The aforementioned spectral data are similar to those reported in the literature for 3-HPA [103,238] (Table 5). Therefore, sample 3A corresponds to 3-HPA (3-hydroxypropionic acid).

**Table 5:** NMR data of isolated compounds from *P*. *cupana* endophytic fungi.

1-Hydroxy-8-methoxyanthraquinone			3-Hydroxypropionic acid			Di-(2-ethylhexyl) phthalate		
Hydrogen	Sample 17A^1^^,^^3^	Ayer et al. (1989)^4^^,^^5^	Hydrogen	Sample 3A^1^^,^^2^	Schwarz et al. (2004)^1^^,^^2^	Hydrogen	Sample 070^1^^,^^2^	Amade et al. (1994)^4^^,^^6^
OH	-	12.96 (1H, s)	H-1	3.07 (2H, t, J = 6.02 Hz)	2.95 (2H, t, J = 6.02 Hz)	1	0.90 (6H, t, J = 6.5 Hz)	0.82 (t, J = 5.3 Hz)
H-5	7.95 (1H, d, J = 7.5)	7.96 (1H, dd, J = 1.3, 7.8 Hz)	H-2	4.68 (2H, t, J = 6.05 Hz)	4.61 (2H, t, J = 6.02 Hz)	2–4	1.28–1.33 (8H, m)	1.15–1.30 (m)
H-1	7.86 (1H, t, J = 7.9)	-	Carbon			5	-	1.59 (m)
H-4	7.77 (1H, d, J = 7.2)	7.77 (1H, dd, J = 1.3, 7.8 Hz)	C-1	174.51	171.7	6	4.20 (2H, qd, J = 5.65, 8.4 Hz)	4.13 (m)
H-6	7.72 (1H, t, J = 7.7)	7.74 (1H, t, J = 7.8 Hz)	C-3	69.29	69.8	8	1.41 (2H, q, J = 6.9 Hz)	1.31 (dq, J = 4.3 Hz)
H-3	7.60 (1H, d, J = 8.1)	7.60 (1H, t, J = 7.8 Hz)	C-2	30.74	30.8	9	0.88 (3H, t, J = 6.4 Hz)	0.79 (t, J = 4.3 Hz)
H-7	7.33 (1H, d, J = 8.5)	7.35 (1H, dd, J = 1.3, 7.8 Hz)				11	7.70 (1H, dd, J = 3.35, 5.80 Hz)	7.60 (dd, J = 6.3; 2.2 Hz)
H-2	-	7.29 (1H, dd, J = 1.3, 7.8 Hz)				12	7.52 (1H, dd, J = 3.35, 5.80 Hz)	7.40 (dd, J = 6.3; 2.2 Hz)
OCH_3_	4.06 (3H, s)	4.04 (3H, s)						
Carbon						Carbon		
C-9	-	188.7				1	10.95	9.91
C-10	-	183.0				2	22.98	21.94
C-1	-	162.6				3	23.74	22.79
C-8	161.95	161.0				4	28.92	27.92
C-10a	135.95	135.9				5	38.73	37.78
C-3	135.72	135.8				6	68.16	67.02
C-6	135.49	135.8				7	167.76	166.58
C-4a	132.79	132.8				8	29.69	29.39
C-4	124.06	124.7				9	14.05	12.96
C-8a	119.36	120.9				10	132.46	131.51
C-5	-	120.2				11	130.88	129.79
C-2	118.62	118.8				12	128.80	127.75
C-7	118.22	118.2						
C-9a	-	117.1						
OCH_3_	55.66	56.7						

Chemical shifts (δ) expressed in ppm.

^**1**^ NMR ^1^H 500 MHz, CDCl_3_

^**2**^ NMR ^13^C 125 MHz, CDCl_3_

^**3**^ NMR ^13^C 125 MHz, CD_3_OD

^**4**^ NMR ^1^H 400 MHz, CDCl_3_

^**5**^ NMR ^13^C 400 MHz, CDCl_3_

^**6**^ NMR ^13^C 100 MHz; CDCl_3_

(-) = Signal not detected in the respective NMR spectrum.

The CH_3_OH subfraction–obtained from the CH_3_OH:H_2_O (7:3) fraction from *D*. *phaseolorum* (8S)–afforded a dark yellow solid sample named 070. Analysis of the ^13^C and ^1^H NMR spectra (Table 5) followed by the HSQC correlation map evidenced the correlation of the signals δH 7.52; 7.70; 4.20; 1.41; 1.28–1.33; 1.25; 0.88; and 0.90 with the signals δC 130.8; 128.8; 68.16; 23.74; 38.7–28.9–22.9; 29.69; 14.05 and 10.96 respectively. The HMBC data analysis evidenced the following fundamental correlations: δH 7.70 with δC 130.8 and 167.7, δH 7.52 with δC 128.8, δH 4.20 with δC 68, δH 1.33–1.28 with δC 22.9 and 23.74. Mass spectrum showed a molecular ion peak at *m/z* = 390. Together, the spectral data indicates that sample 070 corresponds to DEHP (di-(2-ethylhexyl) phthalate) [239].

The CH_3_OH extract fraction obtained from *T*. *asperellum* (1BDA) provided a solid light-yellow sample named 17A after a HPLC purification step. Analyses of ^13^C and ^1^H NMR spectra (Table 5) followed by the HSQC correlation map evidenced the correlation of the signals δH 7.95; 7.86; 7.72; 7.60; 7.33 and 4.06 with the signals δC 118.62; 135.72; 135.49; 118.22; 124.06 and 55.67, respectively. Mass spectrum showed a molecular ion peak at *m/z* = 254. Thus, sample 17A corresponds to 1-hydroxy-8-methoxyanthraquinone, corroborating literature reports [240].

DEHP is used as plasticizer in the plastics industry and is considered as a persistent environmental pollutant due to its extensive usage [241]. DEHP was isolated from endophytic microorganisms [242,243], but not from those belonging to the genus *Diaporthe*. Hence, this is the first report on the production of DEHP by species of the genus *Diaporthe*. As DEHP is present in laboratory equipment and accessories [244,245] and it is considered a common contaminant of analytical equipment [246,247], several authors have questioned whether DEHP is a natural product or an analytical contaminant [248–250]. We believe that DEHP is a fungal product because it was exclusively detected in this sample.

3-HPA is commercially available and used in a range of industrial applications [251]. This compound can be synthesized through different metabolic pathways, from different precursors, and by both prokaryotes and eukaryotes [252], including endophytes [103,238,253]. There are few literature reports on 1-hydroxy-8-methoxyanthraquinone. It was produced through chemical synthesis [254] and isolated for the first time as a natural product from the phytopathogenic fungus *Leptographium wageneri* [240]. To the best of our knowledge, this is the first report on the isolation of 1-hydroxy-8-methoxyanthraquinone from an endophytic fungus and from a microorganism belonging to the genus *Trichoderma*.

### Antibacterial, antitumor and genotoxic activity of the isolated compounds

Here we addressed whether the three special metabolites isolated from endophytic fungal species that colonize *P*. *cupana* display antibacterial, antitumor and genotoxic effecs. First, we used the minimal inhibitory concentration test to examine their antibacterial activity. 3-HPA and DEHP, at all the concentrations tested, exerted the strongest antimicrobial activity against *P*. *aeruginosa* susceptible and multiresistant strains (Table 6). The growth of bacterial colonies in culture medium indicated that 3-HPA and DEHP were bacteriostatic. The antibacterial and nematicidal activity of 3-HPA was previously reported [103,238], but the mechanisms underlying its bactericidal action remain unclear [252]. DEHP exerts antibiotic activity against resistant and susceptible pathogenic bacterial strains [255–259]. In contrast, 1-hydroxy-8-methoxyanthraquinone did not suppress the growth of the bacterial strains tested and there are no literature reports on its antimicrobial activity.

**Table 6:** Minimal inhibitory concentration (μg/mL) of the special metabolites isolated from endophytic fungi of *P*. *cupana*.

Sample	S.A (M)	E.C (M)	P.A (M)	S.A (S)	E.C (S)	P.A (S)
**3-hydroxypropionic acid**	>30	>30	0.23	>30	>30	0.23
**Di-(2-ethylhexyl) phthalate**	>30	>30	0.23	>30	>30	0.23
**Tetracycline**	> 5.000	> 5.000	> 5.000	> 5.000	> 5.000	> 5.000

E.C = *Escherichia coli*; P.A = *Pseudomonas aeruginosa*; S.A = *Staphylococcus aureus*; (M) = Multiresistant strain; (S) = Susceptible strain.

Second, we assessed the antitumor effect of the three special metabolites against CHO and B16F10 cells *in vitro*. 3-HPA reduced the viability of both tumor cell lines in a concentration-dependent manner (S5 Fig). A 72-h treatment with 3-HPA at 100 μg/ml diminished CHO and B16F10 cell viability to 80% and 40%, respectively. 3-HPA is strongly cytotoxic towards prokaryotic cells [252], but there are no reports on its antitumor effect. Our data suggest that 3-HPA is toxic to both tumor and non-tumor cell lines and exerts stronger cytotoxic effect on the former.

There are several reports on the DEHP citotoxicity against tumour and non-tumor cell lines [257,258,260] and its risk to animal health by targeting several organs [261–263]. DEHP was cytotoxic towards CHO and B16F10 cell lines: it decreased their cell viability to 70% and 50%, respectively, after a 72-h treatment at a concentration of 100 μg/ml (S6 Fig). On the other hand, 1-hydroxy-8-methoxyanthraquinone did not affect the viability of both cell lines investigated and there are no literature reports on its antitumor activity.

Third, we assessed the genotoxicity of 3-HPA in V79 cells. 3-HPA diminished the cell viability by less than 20%, even at concentrations as high as 100 μg/ml (S7 Fig); it excludes the possible interference of DNA damage caused by cytotoxic concentrations of the test-sample. 3-HPA did not induce DNA damage (genotoxicity), at least under the conditions assessed (S8 Fig). The cytotoxicity of 3-HPA is well-documented and can be mediated by DNA damage [264,265]. As we did not find information about its genotoxicity in the scientific literature, we recommend performing additional mutagenic tests to examine whether it is safe. 3-HPA was cytotoxic towards CHO cells but not towards V79 cells, corroborating literature reports that these cell lines have different sensibility to cytotoxic compounds, and that CHO cells can be several folds more sensible than V79 cells to toxic molecules [266].

## Conclusion

We isolated and identified thirty-four endophytic fungal species in *P*. *cupana*: eight in seeds, twenty-three in roots and three in both organs. Eight species were not previously reported as endophytic, including *X*. *ganodermophthora*, which was the most abundant species in roots. These findings demonstrate the potential of the Amazonia forest as an environment with high diversity of endophytic fungi. The plant geographic location, clonal type, and organ are factors that influence the structure of the endophytic fungal community of *P*. *cupana*. The plant organ is the most important factor that causes differentiation in the community’s composition. We confirmed that mycorrhizae and DSE colonize susceptible and tolerant genotypes of *P*. *cupana*. *P*. *asparagi* (22TSA) and *P*. *pinodella* (28S) develop microsclerotia on roots under *in vitro* conditions. The endophytic fungal community of *P*. *cupana* harbors species bearing some plant growth-promoting traits, including the synthesis of enzymes, phytohormones and biologically active molecules. We isolated and identified 3-hydroxypropionic acid and di-(2-ethylhexyl)phthalate from *Diaporthe phaseolorum* (8S) and 1-hydroxy-8-methoxyanthraquinone from *Trichoderma asperellum* (1BDA). This study opens possibilities for further investigations, because some species have never been explored for the biological control of crop pests like insects, weeds or parasites, and for the production of chemically active constituents. Hence, they represent potential sources of new and valuable organic molecules.

## Supporting information

S1 Table Classification of the 34 identified endophytic fungal species in *P*. *cupana* seeds and roots into putative ecological roles. (DOCX)

S1 Appendix NMR spectra. (DOCX)

S1 Fig Distribution of endophytic fungal species of *P*. *cupana* within the communities studied. Mu = Manaus; Me = Maués; Su = Susceptible phenotype (CMU 300); To = Tolerant phenotype (CMU 871); Se = Seeds; Ro = Roots.
(TIF)

S2 Fig NMDS plot of the endophytic fungal community structure in *P*. *cupana* using the Jaccard coefficient. Each point represents a single endophytic community. Permutation tests resulted a highly significant classification (*P* = 0.001). The lines separate communities from seeds and roots.
(TIF)

S3 Fig Graphical representation of the network of all the culturable endophytic fungal species isolated from *P*. *cupana* roots and seeds. The size of each node is proportional to its betweeness centrality. Blue, yellow, and red nodes indicate a high, intermediate, and low degree of betweenness centrality, respectively. Thick lines represent positive (Spearman’s ρ>0.6) and significant (*P*<0.05) correlations. Thin lines represent positive non-significant correlations (*P*>0.05).
(TIF)

S4 Fig Modules within the network of culturable endophytic fungal species isolated from *P*. *cupana* roots and seeds. Different colors represent the two modular communities identified. Green and blue modules represent the species isolated from seeds and roots, respectively (modularity index = 0.303, *P* < 0.01).
(TIF)

S5 Fig Viability of Chinese hamster ovary (CHO) and *Mus musculus* skin melanoma (B16F10) cells treated with 3-hydroxypropionic acid (3-HPA) for 72 h. *(CHO) and **#**(B16F10): *P* < 0.05 vs. control (untreated cells); Dunnett's multiple comparison test.
(TIF)

S6 Fig Viability of Chinese hamster ovary (CHO) and *Mus musculus* skin melanoma (B16F10) cells treated with di-(2-ethylhexyl)phthalate (DEHP) for 72 h. *(CHO) and **#**(B16F10): *P* < 0.05 vs. control (untreated cells); Dunnett's multiple comparison test.
(TIF)

S7 Fig Viability (%) of V79 cells treated with 3-hydroxypropionic acid (3-HPA). Negative control: untreated cells. Positive control: 10 μM doxorubicin. *P < 0.05 vs. negative control (Dunnett's multiple comparison test).
(TIF)

S8 Fig DNA damage (% DNA tail) in V79 cells treated with 3-hydroxypropionic acid (3-HPA). Negative control: untreated cells. Positive control: 200 μM hydrogen peroxide. * P < 0.05 vs. negative control (Dunnett's multiple comparison test).
(TIF)
