# Supplementary material for: Diversity of cultivable fungal endophytes in Paullinia cupana (Mart.) Ducke and bioactivity of their secondary metabolites
Source: PLoS One. 2018 Apr 12;13(4):e0195874. doi: 10.1371/journal.pone.0195874 (PMC5897019; doi:10.1371/journal.pone.0195874)
Supplement: S1 Table — (DOCX) [file pone.0195874.s001.docx]

**S1 Table.** Classification of the 34 identified endophytic fungal species in *Paullinia cupana* seeds and roots into putative ecological roles*.

| **Species** | **Putative ecological role** |
| --- | --- |
| ***Xylogone ganodermophthora*** | Mycoparasite [2]. |
| ***Phomopsis asparagi*** | Endophyte [3], Phytopathogen [4]. |
| ***Fusarium oxysporum*** | Endophyte [5], Saprophyte [6], Phytopathogen [7], Mycoparasite [8], Entomopathogen [9], Coprophilous [10]. |
| ***Periconia macrospinosa*** | Endophyte [11], Saprophyte [12]. |
| ***Diaporthe phaseolorum*** | Endophyte [13], Phytopathogen [14]. |
| ***Mariannaea camptospora*** | Saprophyte [15], Coprophilous [10]. |
| ***Diaporthe hongkongensis*** | Endophyte [16], Phytopathogen [17]. |
| ***Trichoderma harzianum*** | Endophyte [18], Saprophyte [19], Mycoparasite, [20], Entomopathogen [21]. |
| ***Humicola fuscoatra*** | Endophyte [22], Saprophyte [23], Phytopathogen [24], Mycoparasite [25]. |
| ***Mycoleptodiscus terrestris*** | Endophyte [26], Phytopathogen [27]. |
| ***Diaporthe melonis*** | Endophyte [28], Phytopathogen [29]. |
| ***Sydowiella fenestrans*** | Endophyte [30]. |
| ***Melanconiella elegans*** | Endophyte [31]. |
| ***Phomopsis lagerstroemiae*** | Endophyte [3]. |
| ***Glomerella acutata*** | Endophyte [32], Phytopathogen [33], Entomopathogen [34]. |
| ***Gibberella zeae*** | Endophyte [35], Saprophyte [36], Phytopathogen [37]. |
| ***Paraphaeosphaeria arecacearum*** | Phytopathogen [27]. |
| ***Nigrograna mackinnonii*** | Endophyte [38]. |
| ***Pestalotiopsis microspora*** | Endophyte [39], Saprophyte [40], Phytopathogen [41]. |
| ***Arxiella dolichandrae*** | Phytopathogen [42]. |
| ***Trichoderma asperellum*** | Endophyte [43], Saprophyte, Mycoparasite [44], Entomopathogen [45]. |
| ***Fusarium polyphialidicum*** | Endophyte [46], Saprophyte [47], Phytopathogen [48], Entomopathogen [49]. |
| ***Diaporthe terebinthifolii*** | Endophyte [13] |
| ***Fusarium solani*** | Endophyte [50], Saprophyte [51], Phytopathogen [52], Entomopathogen [53]. |
| ***Peyronellaea pinodella*** | Phytopathogen [54]. |
| ***Fomitopsis meliae*** | Endophyte [55], Saprophyte [56]. |
| ***Parapleurotheciopsis inaequiseptata*** | Saprophyte [57]. |
| ***Colletotrichum gloeosporioides*** | Endophyte [13], Phytopathogen [58], Saprophyte [59], Mycoparasite [60], Entomopathogen [61]. |
| ***Mycena robusta*** | Saprophyte [62]. |
| ***Penicillium janthinellum*** | Endophyte [63], Phytopathogen [64], Saprophyte [65]. |
| ***Paecilomyces parvisporus*** | Endophyte [66], Entomopathogen [67]. |
| ***Nectria rigidiuscula*** | Endophyte [68], Phytopathogen [69]. |
| ***Talaromyces pinophilus*** | Endophyte[70], Mycoparasite [71]. |

* Classification was based on literature reports and the USDA-ARS database (https://nt.ars-grin.gov/fungaldatabases/fungushost/fungushost.cfm). Full references of the cited papers are given as footnotes. *Pochonia boninensis* is an edaphic species without a well-defined ecological role [1].

# References

1. Nonaka K, Ōmura S, Masuma R, Kaifuchi S, Masuma R. Three new *Pochonia* taxa (*Clavicipitaceae*) from soils in Japan. Mycologia. 2013;105: 1202–1218. doi:10.3852/12-132

2. Kang H-J, Sigler L, Lee J, Gibas CFC, Yun S-H, Lee Y-W. *Xylogone ganodermophthora* sp. nov., an ascomycetous pathogen causing yellow rot on cultivated mushroom *Ganoderma lucidum* in Korea. Mycologia. 2010;102: 1167–84. doi:10.3852/09-304

3. Murali TS, Suryanarayanan TS, Geeta R. Endophytic *Phomopsis* species: host range and implications for diversity estimates. Canadian journal of microbiology. 2006;52: 673–680. doi:10.1139/w06-020

4. Abdelrahman M, Suzumura N, Mitoma M, Matsuo S, Ikeuchi T, Mori M, et al. Comparative de novo transcriptome profiles in  *Asparagus officinalis* and *A. kiusianus* during the early stage of *Phomopsis asparagi* infection. Scientific Reports. 2017;7: 2608. doi:10.1038/s41598-017-02566-7

5. Dababat AE-F, Sikora R. Influence of the mutualistic endophyte *Fusarium oxysporum* 162 on *Meloidogyne incognita* attraction and invasion. Nematology. Brill; 2007;9: 771–776. doi:10.1163/156854107782331225

6. Moslemi A, Ades PK, Groom T, Nicolas ME, Taylor PWJ. *Fusarium oxysporum* and *Fusarium avenaceum* associated with yield-decline of pyrethrum in Australia. European Journal of Plant Pathology. 2017; 1–14. doi:10.1007/s10658-017-1161-5

7. Epstein L, Kaur S, Chang PL, Carrasquilla-garcia N, Lyu G, Cook DR, et al. Races of the celery pathogen *Fusarium oxysporum* f. sp. apii are polyphyletic. Phytopathology. 2017;107: 463–473. doi:10.1094/PHYTO-04-16-0174-R

8. Vajna L. Phytopathogenic *Fusarium oxysporum* Schlecht, as a necrotrophic mycoparasite. Journal of Phytopathology. 1985;114: 338–347. doi:10.1111/j.1439-0434.1985.tb00629.x

9. Prakash S, Singh G, Soni N, Sharma S. Pathogenicity of *Fusarium oxysporum* against the larvae of *Culex quinquefasciatus* (Say) and *Anopheles stephensi* (Liston) in laboratory. Parasitology Research. 2010;107: 651–655. doi:10.1007/s00436-010-1911-1

10. Peterson RA, Bradner JR, Roberts TH, Nevalainen KMH. Fungi from koala (*Phascolarctos cinereus*) faeces exhibit a broad range of enzyme activities against recalcitrant substrates. Letters in Applied Microbiology. 2009;48: 218–225. doi:10.1111/j.1472-765X.2008.02513.x

11. Wearn JA, Sutton BC, Morley NJ, Gange AC. Species and organ specificity of fungal endophytes in herbaceous grassland plants. Journal of Ecology. 2012;100: 1085–1092. doi:10.1111/j.1365-2745.2012.01997.x

12. Elmholt S, Kjøller A. Comparison of the Occurrence of the Saprophyte Soil Fungi in Two Differently Cultivated Field Soils. Biological Agriculture & Horticulture. Taylor & Francis Group ; 1989;6: 229–239. doi:10.1080/01448765.1989.9754520

13. Ferreira MC, Vieira M de LA, Zani CL, Alves TM de A, Junior PAS, Murta SMF, et al. Molecular phylogeny, diversity, symbiosis and discover of bioactive compounds of endophytic fungi associated with the medicinal Amazonian plant *Carapa guianensis* Aublet ( *Meliaceae*). Biochemical Systematics and Ecology. 2015;59: 36–44. doi:10.1016/j.bse.2014.12.017

14. Grijalba P, Ridao A del C. Survival of *Diaporthe phaseolorum* var. caulivora (causal agent of soybean stem canker) artificially inoculated in different crop residues. Tropical Plant Pathology. 2012;37: 271–274. doi:10.1590/S1982-56762012000400006

15. Fukuda T, Sudoh Y, Tsuchiya Y, Okuda T, Fujimori F, Igarashi Y. Marianins A and B, prenylated phenylpropanoids from *Mariannaea camptospora*. Journal of Natural Products. 2011;74: 1327–1330. doi:10.1021/np200035m

16. Huang F, Udayanga D, Wang X, Hou X, Mei X, Fu Y, et al. Endophytic *Diaporthe* associated with Citrus: A phylogenetic reassessment with seven new species from China. Fungal Biology. 2015;119: 331–347. doi:10.1016/j.funbio.2015.02.006

17. Akinsanmi OA, Drenth A. Characterisation of husk rot in macadamia. Annals of Applied Biology. 2017;170: 104–115. doi:10.1111/aab.12320

18. Bailey BA, Strem MD, Wood D. *Trichoderma* species form endophytic associations within *Theobroma cacao* trichomes. Mycological Research. 2009;113: 1365–1376. doi:10.1016/j.mycres.2009.09.004

19. Rashmi S, Maurya S, Upadhyay RS. The improvement of competitive Saprophyte capabilities of *Trichoderma* species through the use of chemical mutagens. Brazilian Journal of Microbiology. 2016;47: 10–17. doi:10.1016/j.bjm.2015.11.003

20. da Mota PR, Ribeiro MS, de Castro Georg R, Silva GR, de Paula RG, Silva R do N, et al. Expression analysis of the α-1,2-mannosidase from the mycoparasitic fungus *Trichoderma harzianum*. Biological Control. 2016;95: 1–4. doi:10.1016/j.biocontrol.2015.12.013

21. Sundaravadivelan C, Padmanabhan MN. Effect of mycosynthesized silver nanoparticles from filtrate of *Trichoderma harzianum* against larvae and pupa of dengue vector *Aedes aegypti* L. Environmental Science and Pollution Research. 2014;21: 4624–4633. doi:10.1007/s11356-013-2358-6

22. Krishnamurthy YL, Naik SB, Jayaram S. Fungal communities in herbaceous medicinal plants from the malnad region, southern India. Microbes and Environments. 2008;23: 24–28. doi:10.1264/jsme2.23.24

23. Moharram AM, Bagy MMK, Abdel-Mallek AY. Saprophyte fungi isolated from animal and bird pens in Egypt. Journal of Basic Microbiology. 1987;27: 361–367. doi:10.1002/jobm.3620270706

24. Mohammad T, Attia H. Eco-friendly oil-in-water emulsion formulation of *Eucalyptus* oil for controlling some important phytopathogenic fungi. American Journal of Experimental Agriculture. 2015;9: 1–10. doi:10.9734/AJEA/2015/18262

25. Joshi BK, Gloer JB, Wicklow DT. Bioactive natural products from a sclerotium-colonizing isolate of *Humicola fuscoatra*. Journal of Natural Products. 2002;65: 1734–1737. doi:10.1021/np020295p

26. Ortega HE, Graupner PR, Asai Y, Tendyke K, Qiu D, Shen YY, et al. Mycoleptodiscins A and B, cytotoxic alkaloids from the endophytic fungus *Mycoleptodiscus* sp. F0194. Journal of Natural Products. 2013;76: 741–744. doi:10.1021/np300792t

27. Fry WE. Association of Formamide Hydro-lyase with Fungal Pathogenicity to Cyanogenic Plants. Phytopathology. 1977;77: 1001. doi:10.1094/Phyto-67-1001

28. dos Santos TT, de Souza Leite T, de Queiroz CB, de Araújo EF, Pereira OL, de Queiroz MV. High genetic variability in endophytic fungi from the genus *Diaporthe* isolated from common bean ( *Phaseolus vulgaris* L.) in Brazil. Journal of Applied Microbiology. 2016;120: 388–401. doi:10.1111/jam.12985

29. Beraha L, O’Brien MJ. *Diaporthe melonis* new species a new soft rot of market cantaloupes. Journal of Phytopathology. 1979;94: 199–207. doi:10.1111/j.1439-0434.1979.tb01551.x

30. Du Z, Hyde KD, Yang Q, Liang YM, Tian CM. *Melansporellaceae*: A novel family of *Diaporthales* (Ascomycota). Phytotaxa. 2017;305: 191–200. doi:10.11646/phytotaxa.305.3.6

31. Parpinelli B, Siqueira K, Kellner Filho L, Pimenta L, da Costa R, Parreira R, et al. Effect of endophytic fungal associations on the chemical profile of in vitro *Vochysia divergens* seedlings. Journal of the Brazilian Chemical Society. 2017; 1–7. doi:10.21577/0103-5053.20170091

32. Vieira MLA, Hughes AFS, Gil VB, Vaz ABM, Alves TMA, Zani CL, et al. Diversity and antimicrobial activities of the fungal endophyte community associated with the traditional Brazilian medicinal plant *Solanum cernuum* Vell. (*Solanaceae*). Canadian Journal of Microbiology. 2012;58: 54–66. doi:10.1139/w11-105

33. Ploetz R. Diseases of tropical fruit crops. In: Ploetz RC, editor. Diseases of tropical fruit crops. Wallingford: CABI; 2003. pp. 327–364. doi:10.1079/9780851993904.0000

34. Marcelino J, Giordano R, Gouli S, Gouli V, Parker BL, Skinner M, et al. *Colletotrichum acutatum* var. fioriniae (teleomorph: *Glomerella acutata* var. fioriniae var. nov.) infection of a scale insect. Mycologia. 2008;100: 353–374. doi:10.3852/07-174R

35. Pimenta RS, da Silva JFM, Buyer JS, Janisiewicz WJ. Endophytic fungi from plums (*Prunus domestica*) and their antifungal activity against *Monilinia fructicola*. Journal of food protection. 2012;75: 1883–9. doi:10.4315/0362-028X.JFP-12-156

36. Bujold I, Paulitz TC, Carisse O. Effect of  *Microsphaeropsis* sp. on the production of perithecia and ascospores of *Gibberella zeae*. Plant Disease. 2001;85: 977–984. doi:10.1094/PDIS.2001.85.9.977

37. Cumagun CJR, Bowden RL, Jurgenson JE, Leslie JF, Miedaner T. Genetic mapping of pathogenicity and aggressiveness of *Gibberella zeae* (*Fusarium graminearum*) toward wheat. Phytopathology. 2004;94: 520–526. doi:10.1094/PHYTO.2004.94.5.520

38. Shaw JJ, Spakowicz DJ, Dalal RS, Davis JH, Lehr NA, Dunican BF, et al. Biosynthesis and genomic analysis of medium-chain hydrocarbon production by the endophytic fungal isolate *Nigrograna mackinnonii* E5202H. Applied Microbiology and Biotechnology. 2015;99: 3715–3728. doi:10.1007/s00253-014-6206-5

39. Strobel G, Yang X, Sears J, Kramer R, Sidhu RS, Hess WM. Taxol from *Pestalotiopsis microspora*, an endophytic fungus of *Taxus wallachiana*. Microbiology. Microbiology Society; 1996;142: 435–440. doi:10.1099/13500872-142-2-435

40. Niu X, Hao X, Hong Z, Chen L, Yu X, Zhu X. A Putative Histone Deacetylase Modulates the Biosynthesis of Pestalotiollide B and Conidiation in *Pestalotiopsis microspora*. Journal of Microbiology and Biotechnology. 2015;25: 579–588. doi:10.4014/jmb.1409.09067

41. Zhang C, Liu Y, Wu H, Xu BC, Sun P, Xu Z. Baseline sensitivity of *Pestalotiopsis microspora*, which causes black spot disease on Chinese hickory (Carya cathayensis), to pyraclostrobin. Crop Protection. 2012;42: 256–259. doi:10.1016/j.cropro.2012.07.018

42. Crous PW, Shivas RG, Quaedvlieg W, van der Bank M, Zhang Y, Summerell BA, et al. Fungal Planet description sheets: 214–280. Persoonia - Molecular Phylogeny and Evolution of Fungi. 2014;32: 184–306. doi:10.3767/003158514X682395

43. Hanada RE, Pomella AW V, Costa HS, Bezerra JL, Loguercio LL, Pereira JO. Endophytic fungal diversity in *Theobroma cacao* (cacao) and *T. grandiflorum* (cupuaçu) trees and their potential for growth promotion and biocontrol of black-pod disease. Fungal Biology. 2010;114: 901–910. doi:10.1016/j.funbio.2010.08.006

44. Tchameni SN, Ngonkeu MEL, Begoude BAD, Wakam Nana L, Fokom R, Owona AD, et al. Effect of *Trichoderma asperellum* and arbuscular mycorrhizal fungi on cacao growth and resistance against black pod disease. Crop Protection. 2011;30: 1321–1327. doi:10.1016/j.cropro.2011.05.003

45. Zhuang J, Su L, Wei H, Xiao NW, Li JS, Hua HX, et al. Effects of elevated CO_2_ on the development and physiological metabolic activities of *Nilaparvata lugens* in response to the infection of *Trichoderma asperellum*. International Journal of Pest Management. 2015;61: 292–298. doi:10.1080/09670874.2015.1046970

46. Pan J, Lin Y, Tan N, Gu Y. Cu(II): a “signaling molecule” of the mangrove endophyte *Fusarium oxysporum* ZZF51? BioMetals. Springer Netherlands; 2010;23: 1053–1060. doi:10.1007/s10534-010-9350-0

47. Onyike NBN, Nelson PE. The distribution of *Fusarium* species in soils planted to millet and sorghum in Lesotho, Nigeria and Zimbabwe. Mycopathologia. 1993;121: 105–114. doi:10.1007/BF01103578

48. Barik BP, Tayung K, Jagadev PN. Molecular phylogeny and RNA secondary structure of *Fusarium* species with different lifestyles. Plant Pathology. 2011; 205–219. doi:10.5943/ppq/1/2/5

49. Hajek AE, Elkinton JS, Humber R a. Entomopathogenic hyphomycetes associated with gypsy moth larvae. Mycologia. 1997;89: 825. doi:10.2307/3761102

50. Venugopalan A, Srivastava S. Enhanced camptothecin production by ethanol addition in the suspension culture of the endophyte, *Fusarium solani*. Bioresource Technology. Elsevier; 2015;188: 251–257. doi:10.1016/j.biortech.2014.12.106

51. Taveira GB, Mello ÉO, Carvalho AO, Regente M, Pinedo M, de La Canal L, et al. Antimicrobial activity and mechanism of action of a thionin-like peptide from *Capsicum annuum* fruits and combinatorial treatment with fluconazole against *Fusarium solani*. Biopolymers. 2017;108: e23008. doi:10.1002/bip.23008

52. Mnif I, Hammami I, Triki MA, Azabou MC, Ellouze-Chaabouni S, Ghribi D. Antifungal efficiency of a lipopeptide biosurfactant derived from *Bacillus subtilis* SPB1 versus the phytopathogenic fungus, *Fusarium solani*. Environmental Science and Pollution Research. 2015;22: 18137–18147. doi:10.1007/s11356-015-5005-6

53. Majumdar A, Boetel MA, Jaronski ST. Discovery of *Fusarium solani* as a naturally occurring pathogen of sugarbeet root maggot (Diptera: *Ulidiidae*) pupae: Prevalence and baseline susceptibility. Journal of Invertebrate Pathology. 2008;97: 1–8. doi:10.1016/j.jip.2007.05.003

54. Marinelli E, Orzali L, Lotti E, Riccioni L. Activity of some essential oils against pathogenic seed borne fungi on legumes. Asian Journal of Plant Pathology. 2012;6: 66–74. doi:10.3923/ajppaj.2012.66.74

55. Pinruan U, Rungjindamai N, Choeyklin R, Lumyong S, Hyde KD, Jones EBG. Occurrence and diversity of basidiomycetous endophytes from the oil palm, *Elaeis guineensis* in Thailand. Fungal Diversity. 2010;41: 71–88. doi:10.1007/s13225-010-0029-1

56. Glen M, Yuskianti V, Puspitasari D, Francis A, Agustini L, Rimbawanto A, et al. Identification of basidiomycete fungi in Indonesian hardwood plantations by DNA barcoding. Forest Pathology. 2014;44: 496–508. doi:10.1111/efp.12146

57. Rajeshkumar KC, Crous PW, Groenewald JZ, Seifert KA. Resolving the phylogenetic placement of *Porobeltraniella* and allied genera in the *Beltraniaceae*. Mycological Progress. 2016;15: 1119–1136. doi:10.1007/s11557-016-1234-4

58. Nesher I, Barhoom S, Sharon A. Cell cycle and cell death are not necessary for appressorium formation and plant infection in the fungal plant pathogen *Colletotrichum gloeosporioides*. BMC Biology. 2008;6: 9. doi:10.1186/1741-7007-6-9

59. Barhoom S, Sharon A. Bcl-2 proteins link programmed cell death with growth and morphogenetic adaptations in the fungal plant pathogen *Colletotrichum gloeosporioides*. Fungal Genetics and Biology. 2007;44: 32–43. doi:10.1016/j.fgb.2006.06.007

60. Singh UP, Vishwakarma SN, Basuchaudhury KC. *Acremonium sordidulum* Mycoparasitic on *Colletotrichum dematium* f. truncata in India. Mycologia. Taylor & Francis, Ltd.; 1978;70: 453. doi:10.2307/3759043

61. Marcelino J a P, Gouli S, Parker BL, Skinner M, Schwarzberg L, Giordano R. Host plant associations of an entomopathogenic variety of the fungus, *Colletotrichum acutatum*, recovered from the elongate hemlock scale, *Fiorinia externa*. Journal of insect science (Online). 2009;9: 25. doi:10.1673/031.009.1301

62. Sanville C. The diversity of litter-decomposing fungi in riparian Sitka spruce forests. Humboldt State University. 2000. doi:10211.3/127636

63. Rosa LH, Vieira MLA, Cota BB, Johann S, Alves TMA, Zani CL, et al. Endophytic fungi of tropical forests : A promising source of bioactive prototype molecules for the treatment of neglected diseases. Drug Development - A Case Study Based Insight into Modern Strategies. 2011. pp. 469–486. doi:10.5772/27783

64. Al-Mazroui SS, Al-Sadi AM. 454 pyrosequencing and direct plating reveal high fungal diversity and dominance by Saprophyte species in organic compost. International Journal of Agriculture and Biology. 2016;18: 98–102. doi:10.17957/IJAB/15.0068

65. Mdee LK, Masoko P, Eloff JN. The activity of extracts of seven common invasive plant species on fungal phytopathogens. South African Journal of Botany. 2009;75: 375–379. doi:10.1016/j.sajb.2009.02.003

66. Premalatha K, Gokul S, Kumar A, Mishra P, Mishra P, Ravikumar K, et al. Molecular profiling of fungal assemblages in the healthy and infected roots of *Decalepis arayalpathra* (J. Joseph & V. Chandras) Venter, an endemic and endangered ethnomedicinal plant from Western Ghats, India. Annals of Microbiology. 2015;65: 785–797. doi:10.1007/s13213-014-0919-7

67. Colmenarez Y, Moore D, Polar P, Vasquez C. Population trends of the red palm mite, *Raoiella indica* hirst (Acari: Tenuipalpidae) and associated entomopathogenic fungi in trinidad, antigua, st kitts and nevis and dominica. Acarologia. 2014;54: 433–442. doi:10.1051/acarologia/20142141

68. Yunianto P, Rosmalawati S, Rachmawati I, Priyono Suwarso W, Sumaryono W. Isolation and identification of endophytic fungi from Srikaya plants (*Annona squamosa*) having potential secondary metabolites as anti-breast cancer activity. Microbiology Indonesia. 2012;6: 23–29. doi:10.5454/mi.6.1.4

69. Horst RK. Galls. Westcott’s Plant Disease Handbook. Dordrecht: Springer Netherlands; 2013. pp. 191–193. doi:10.1007/978-94-007-2141-8_30

70. Krishnapura PR, Belur PD. Partial purification and characterization of L-asparaginase from an endophytic *Talaromyces pinophilus* isolated from the rhizomes of *Curcuma amada*. Journal of Molecular Catalysis B: Enzymatic. 2016;124: 83–91. doi:10.1016/j.molcatb.2015.12.007

71. De Stefano S, Nicoletti R, Milone A, Zambardino S. 3-o-methylfunicone, a fungitoxic metabolite produced by the fungus *Penicillium pinophilum*. Phytochemistry. 1999;52: 1399–1401. doi:10.1016/S0031-9422(99)00320-9
