# Supplementary material for: Diversity of cultivable fungal endophytes in Paullinia cupana (Mart.) Ducke and bioactivity of their secondary metabolites
Source: PLoS One. 2018 Apr 12;13(4):e0195874. doi: 10.1371/journal.pone.0195874 (PMC5897019; doi:10.1371/journal.pone.0195874)
Supplement: S1 Appendix — (DOCX) [file pone.0195874.s002.docx]

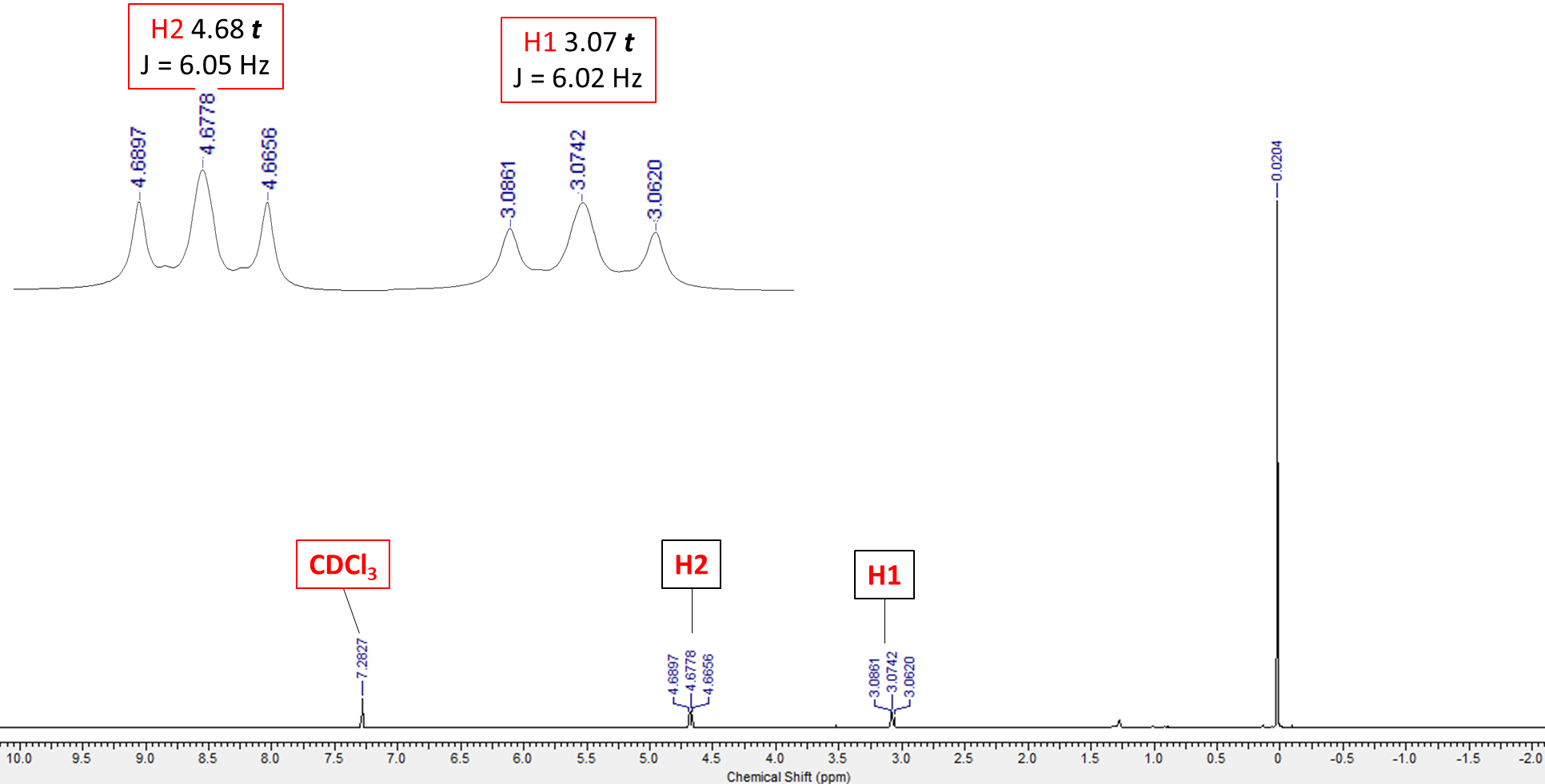


**^1^H NMR sample 3A (CDCl_3_. 500 MHz)**


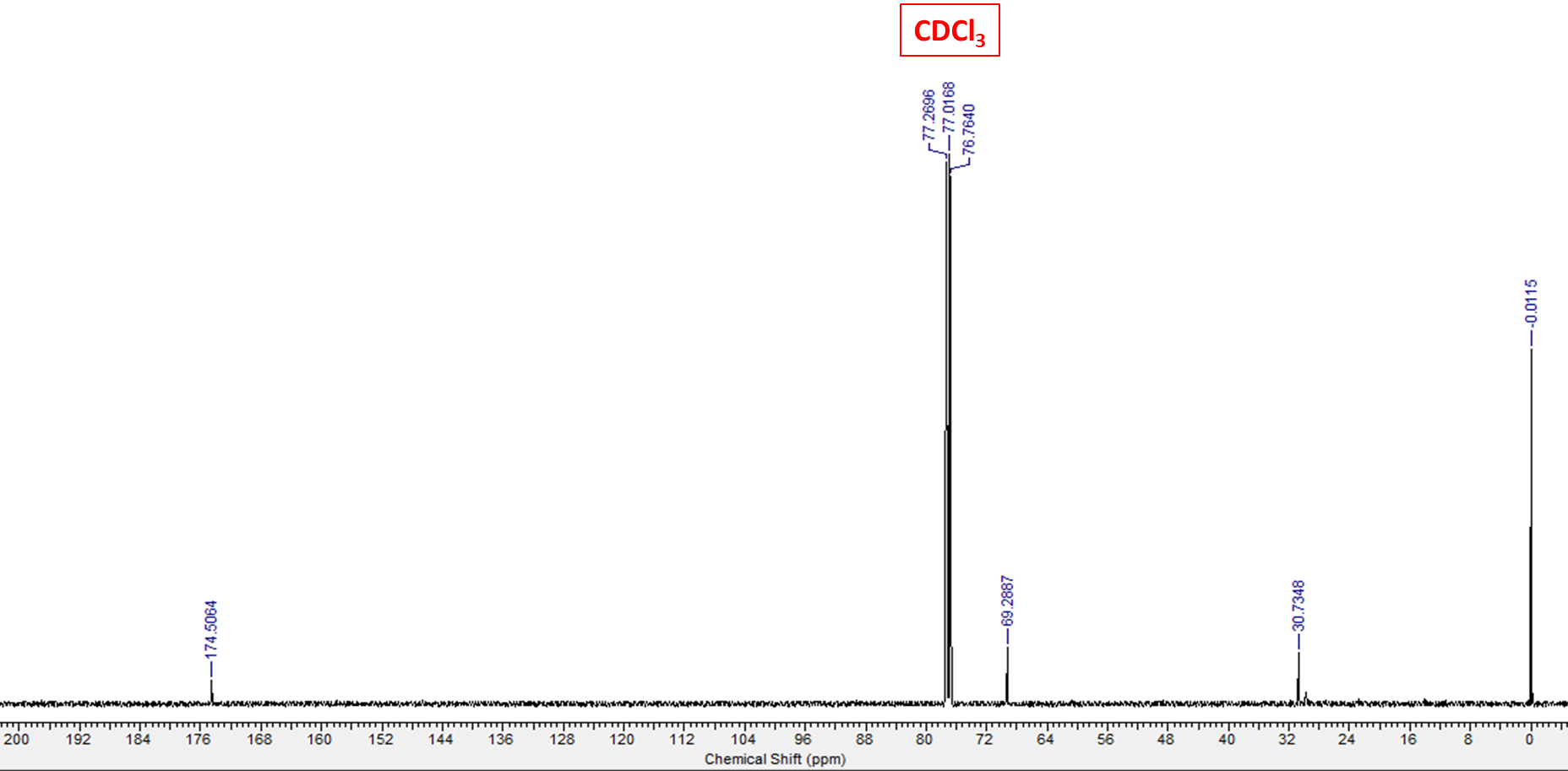


**^13^C NMR sample 3A (CDCl_3_. 125 MHz)**


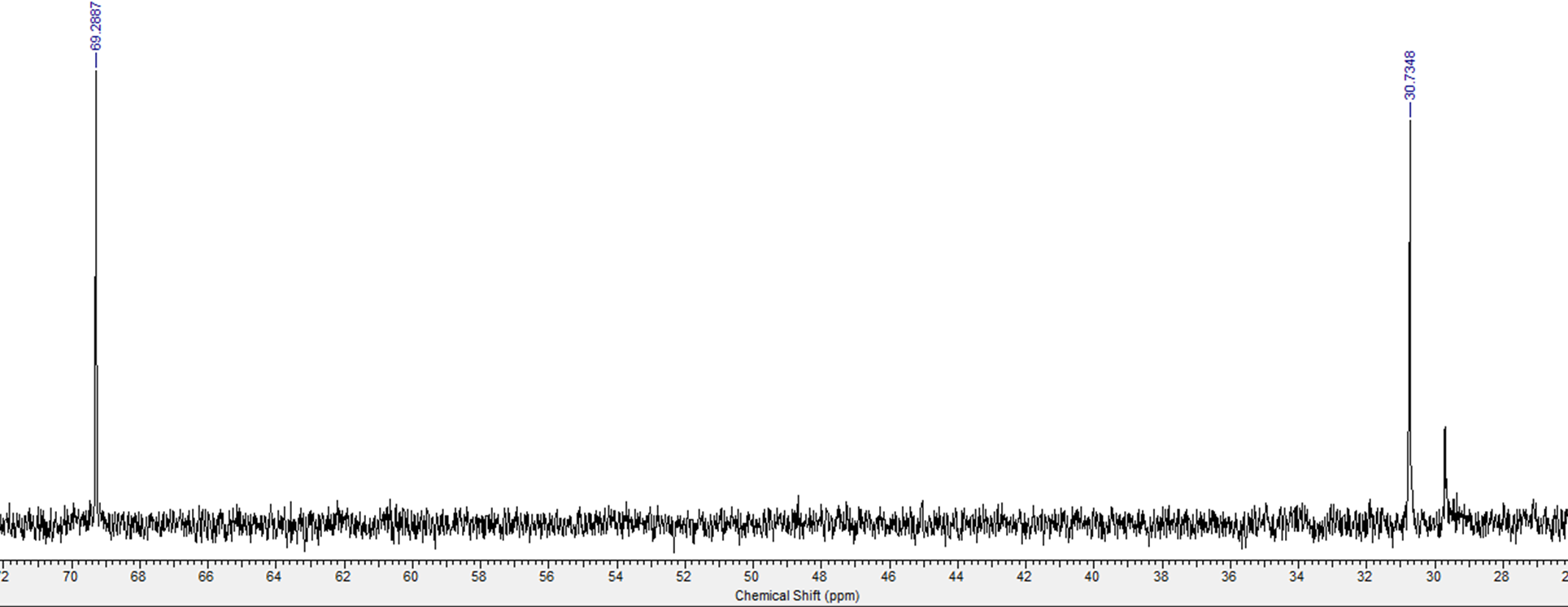


**^13^C NMR sample 3A Expansion I (CDCl_3_. 125 MHz)**


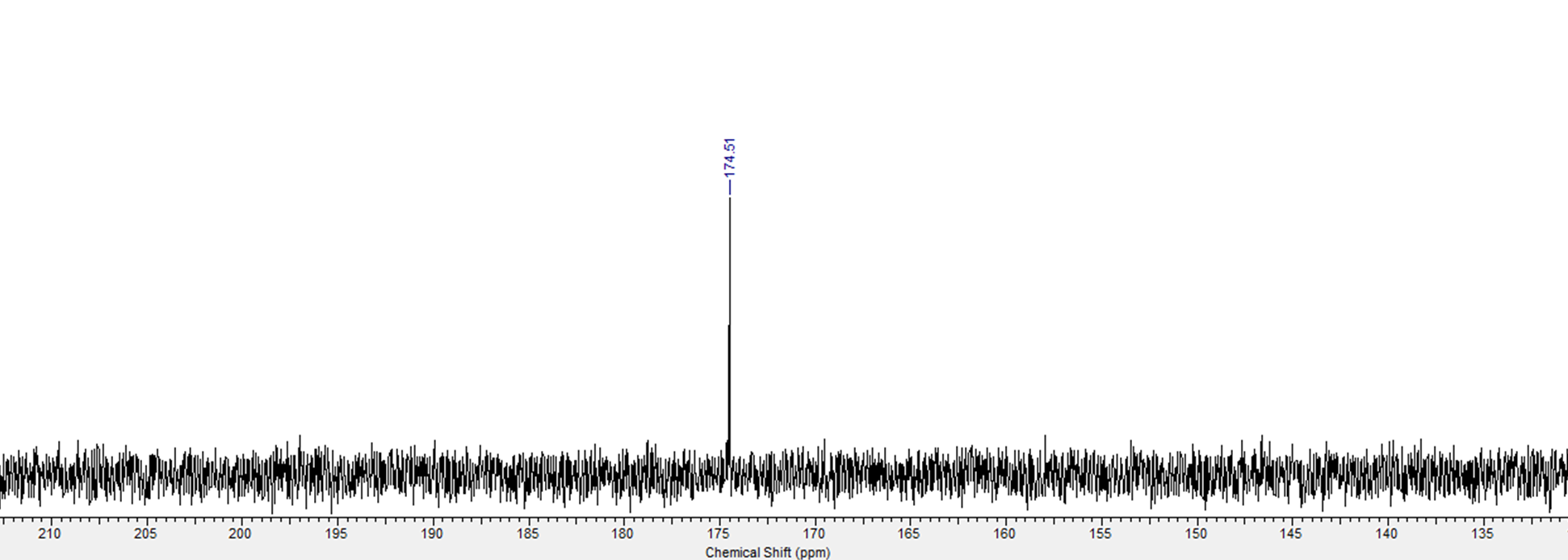


**^13^C sample 3A Expansion II (CDCl_3_. 125 MHz)**


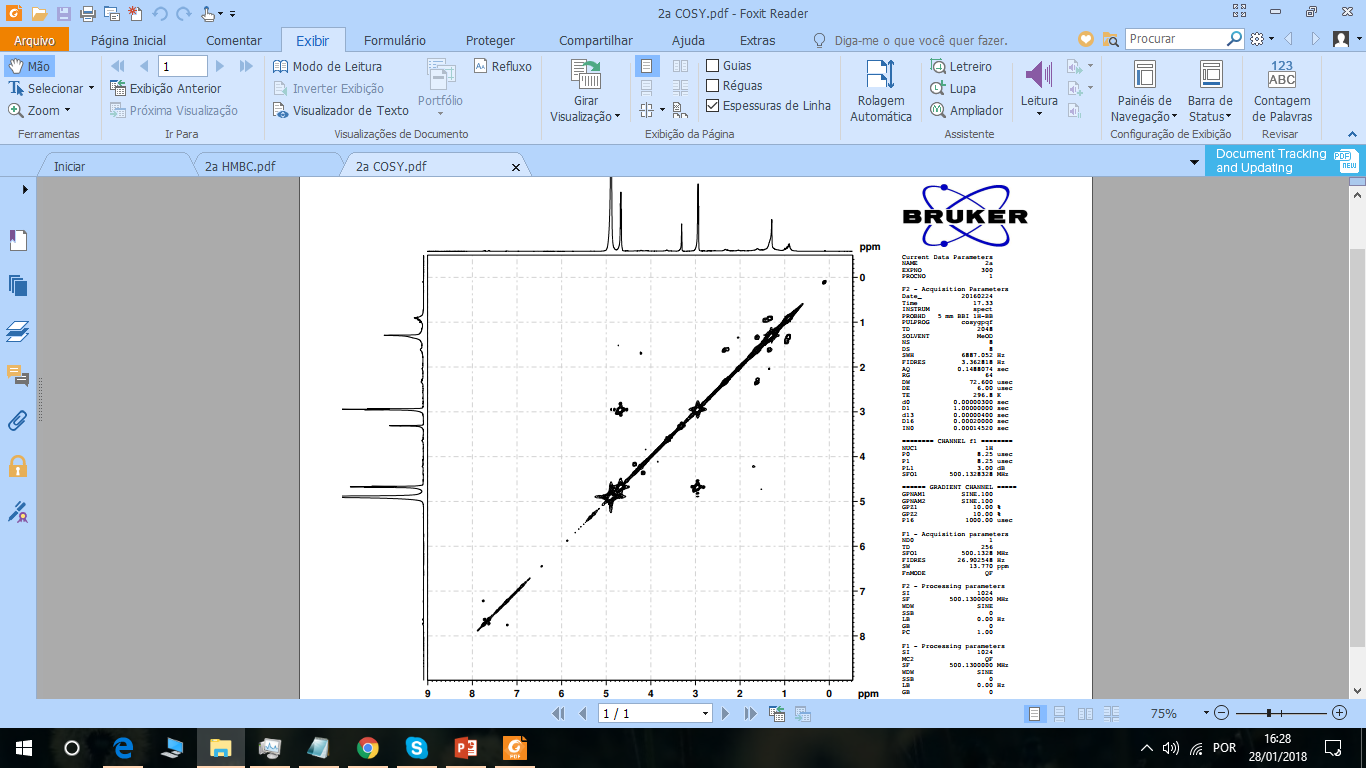

**COSY sample 3A (500 MHz. CDCl_3_)**


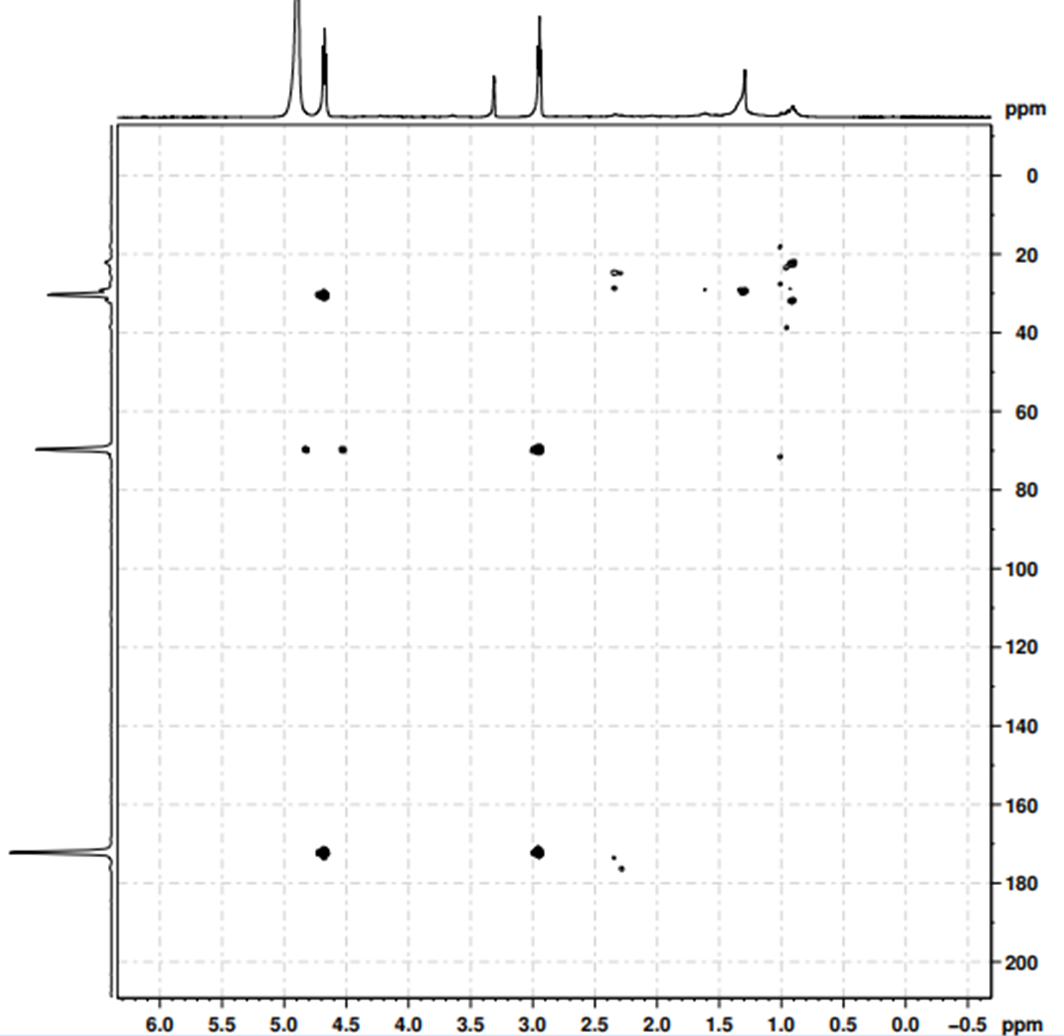

**HMBC sample 3A (500 MHz - ^1^H. 125 MHz - ^13^C. CDCl_3_)**


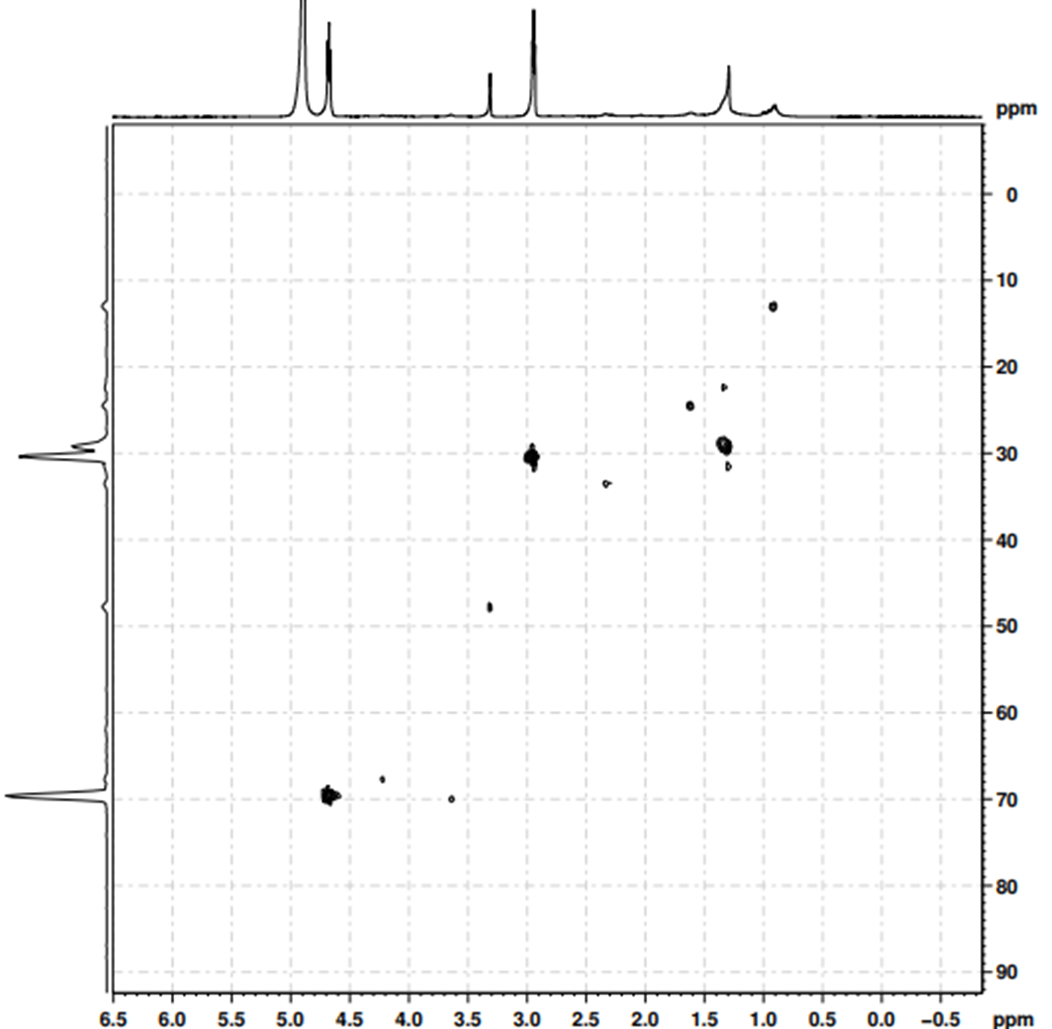

**HSQC sample 3A (500 MHz - ^1^H. 125 MHz - ^13^C. CDCl_3_)**


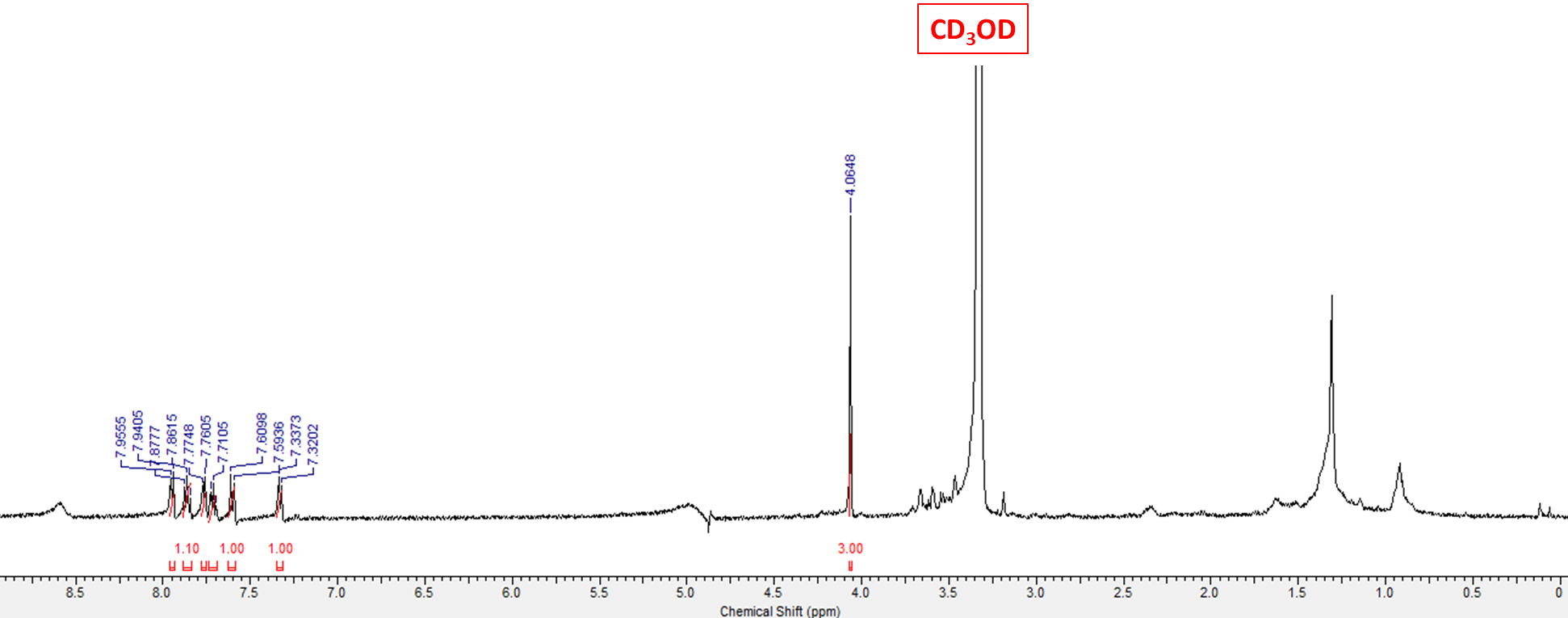


**^1^H NMR sample 17A (CD_3_OD. 500 MHz)**


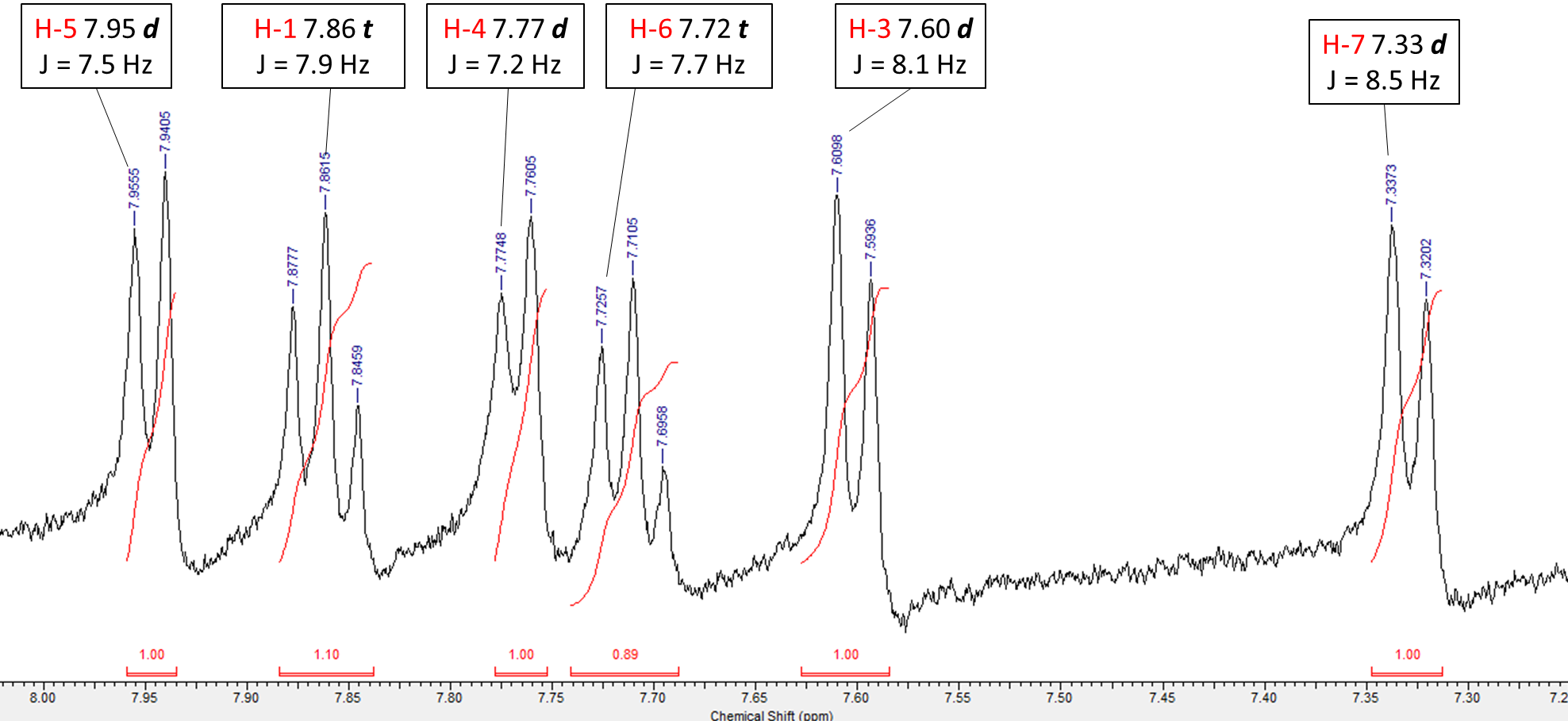


**^1^H NMR sample 17A Expansion I (CD_3_OD. 125 MHz)**


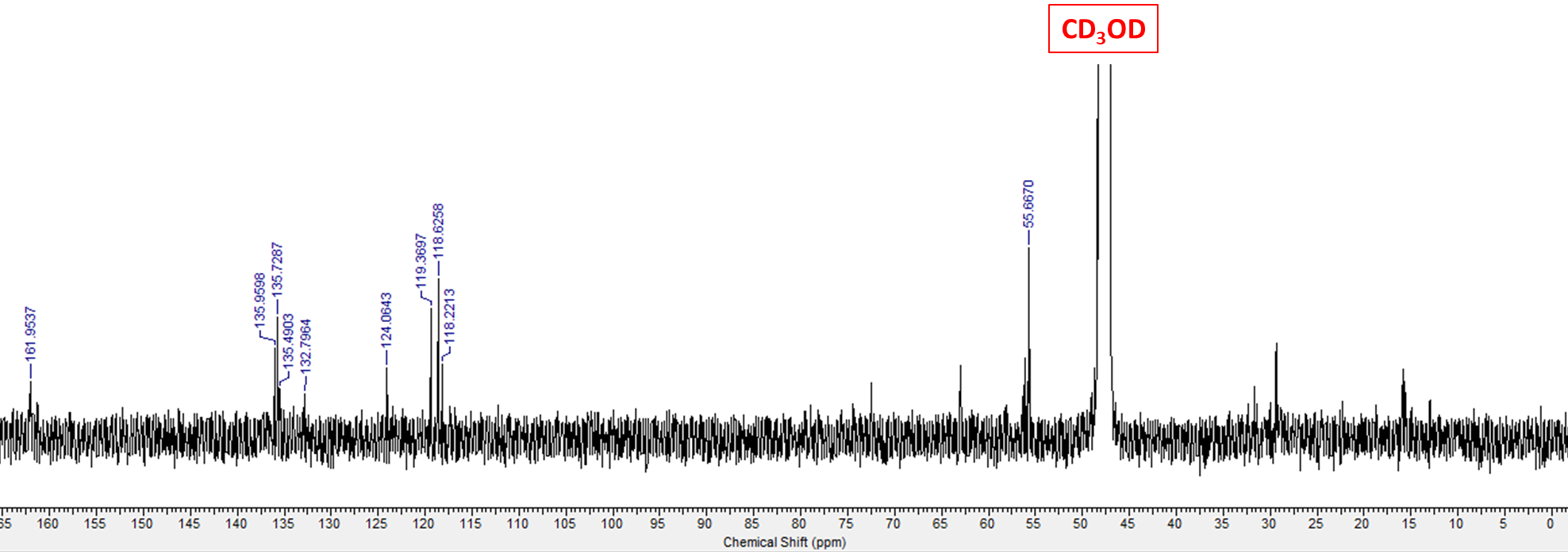


**^13^C NMR sample 17A (CD_3_OD. 125 MHz)**


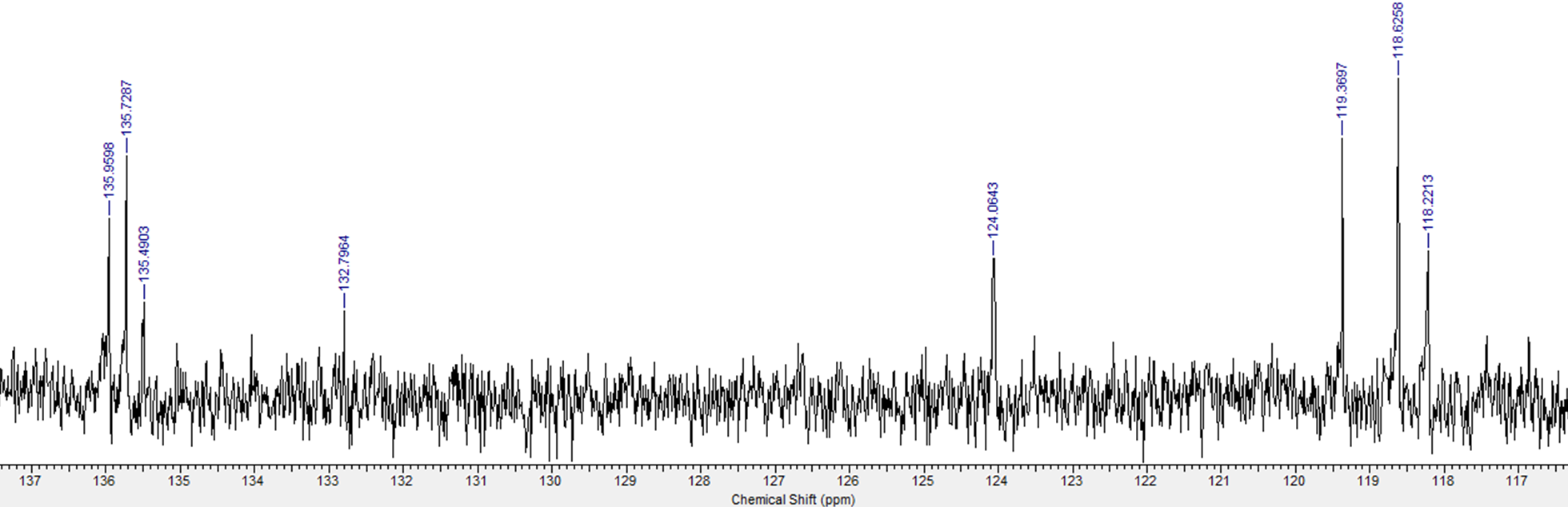


**^13^C NMR sample 17A Expansion I (CD_3_OD. 125 MHz)**


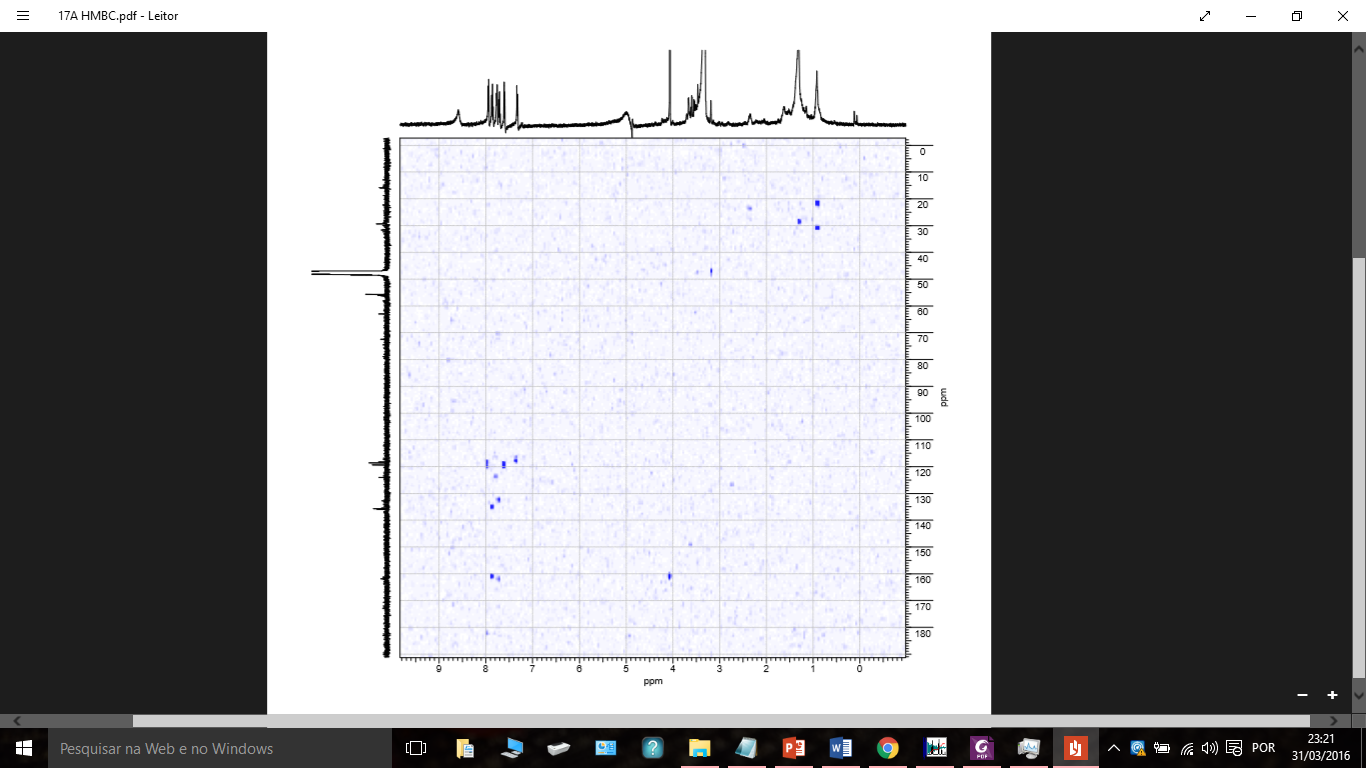

**HMBC sample 17A (500 MHz - ^1^H. 125 MHz - ^13^C. CDCl_3_)**


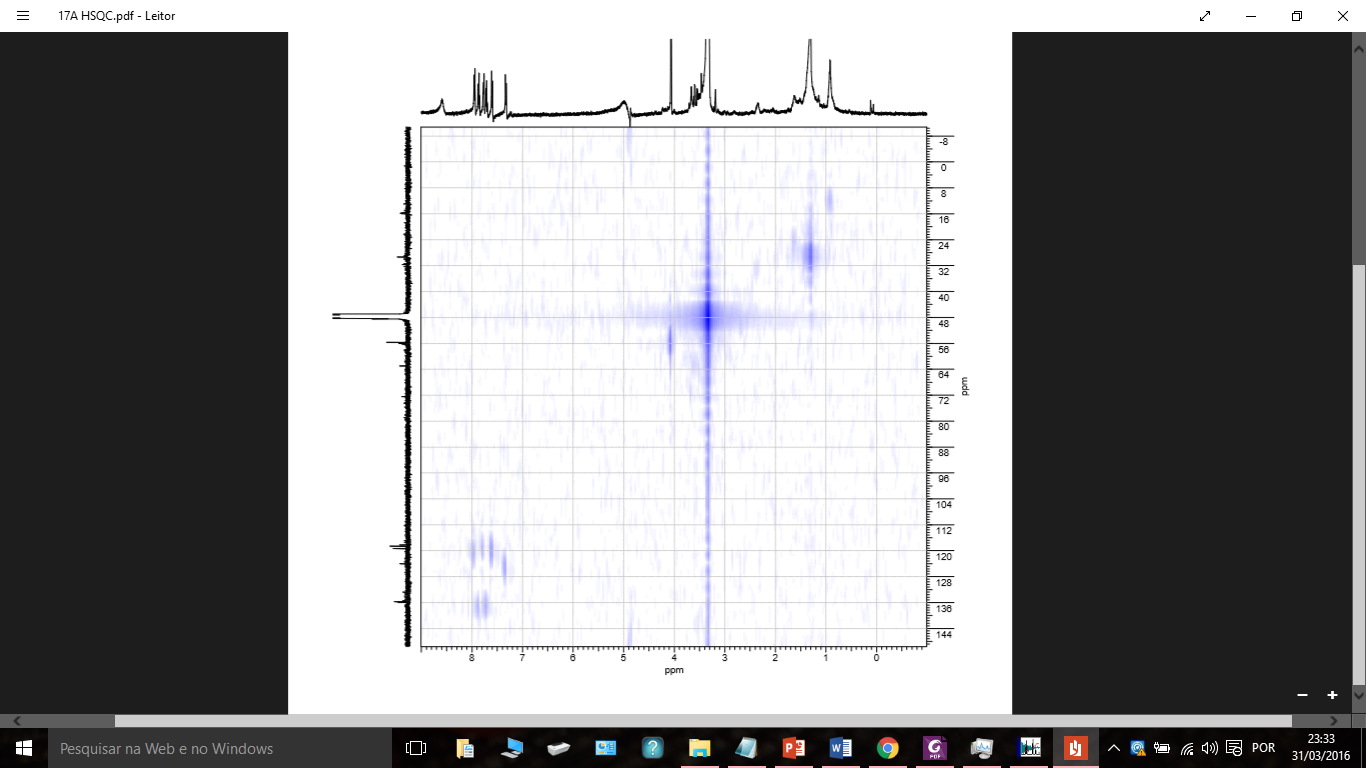

**HSQC sample 17A (500 MHz - ^1^H. 125 MHz - ^13^C. CDCl_3_)**


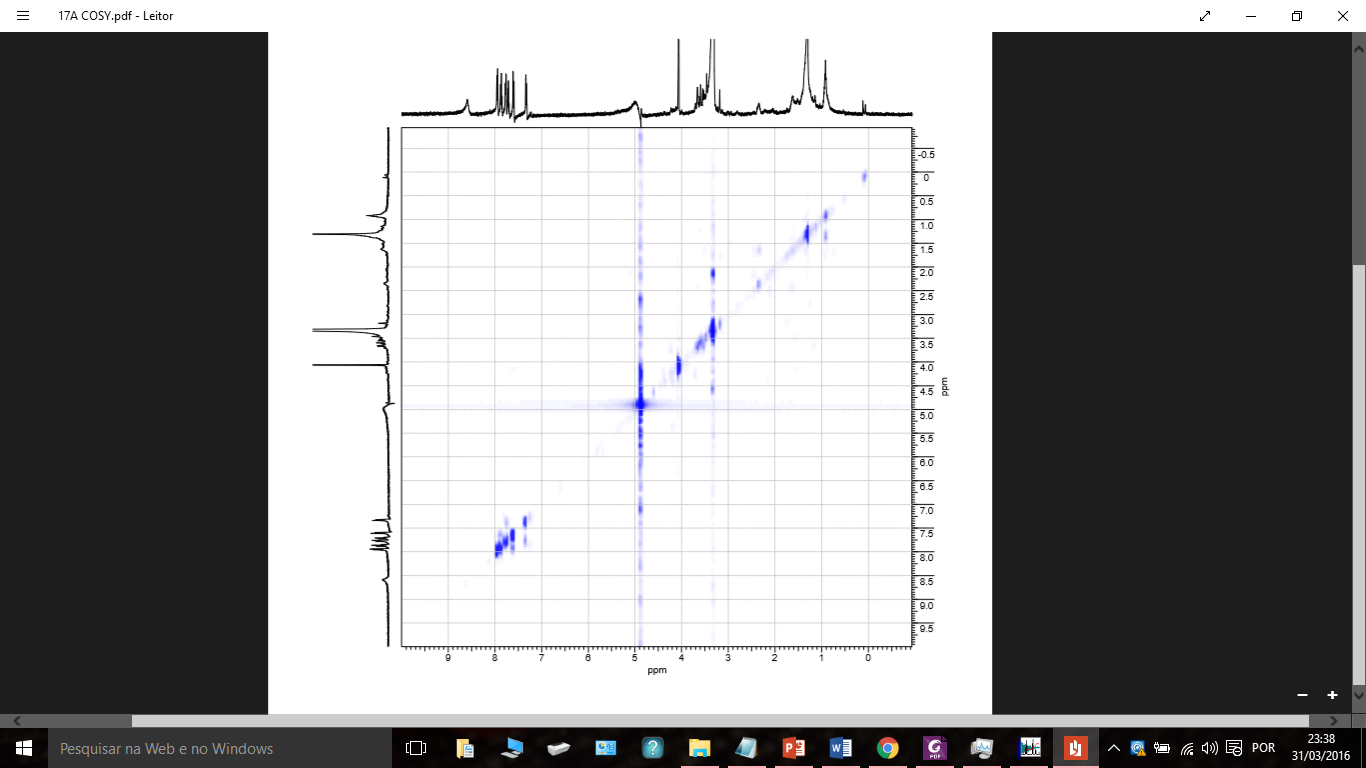

**COSY sample 17A (500 MHz. CDCl_3_)**


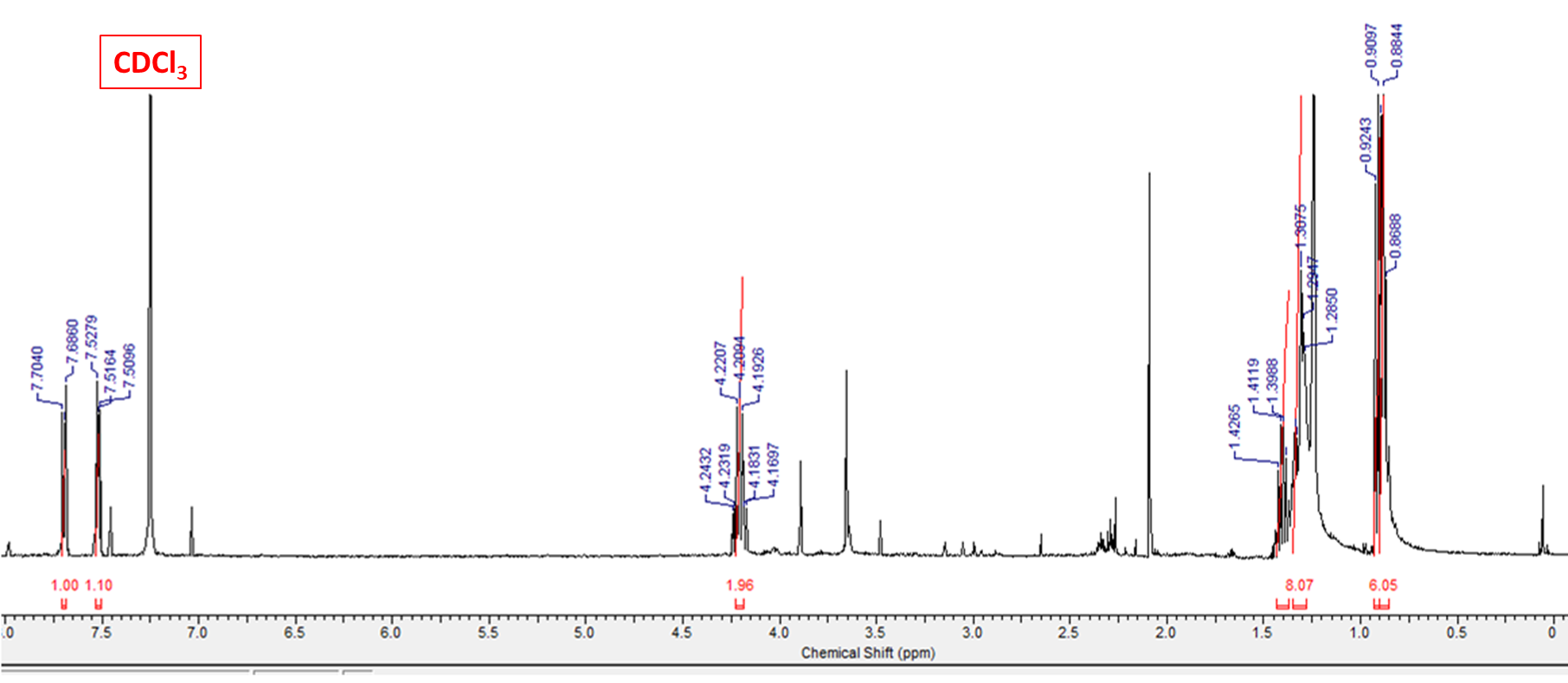


**^1^H NMR sample 070 (CDCl_3_. 500 MHz)**


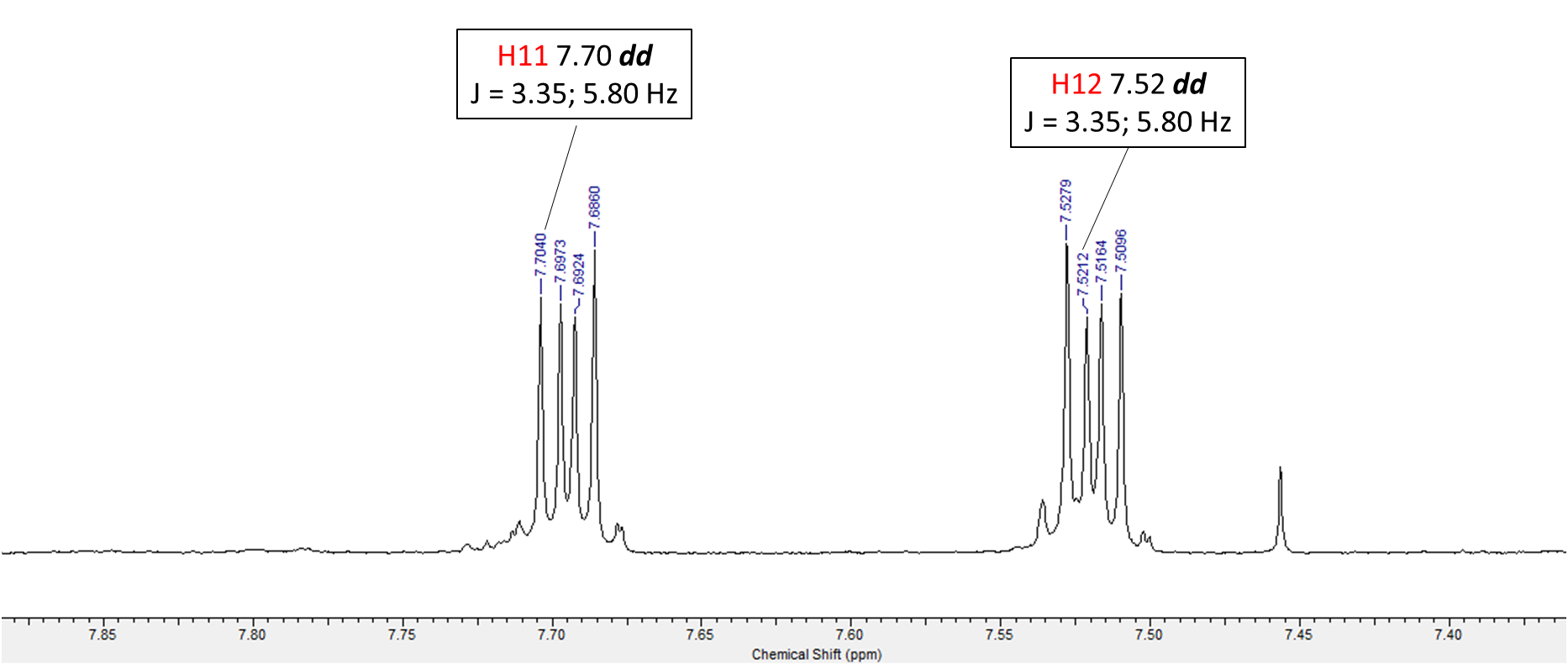


**^1^H NMR sample 070 Expansion I (CDCl_3_. 500 MHz)**


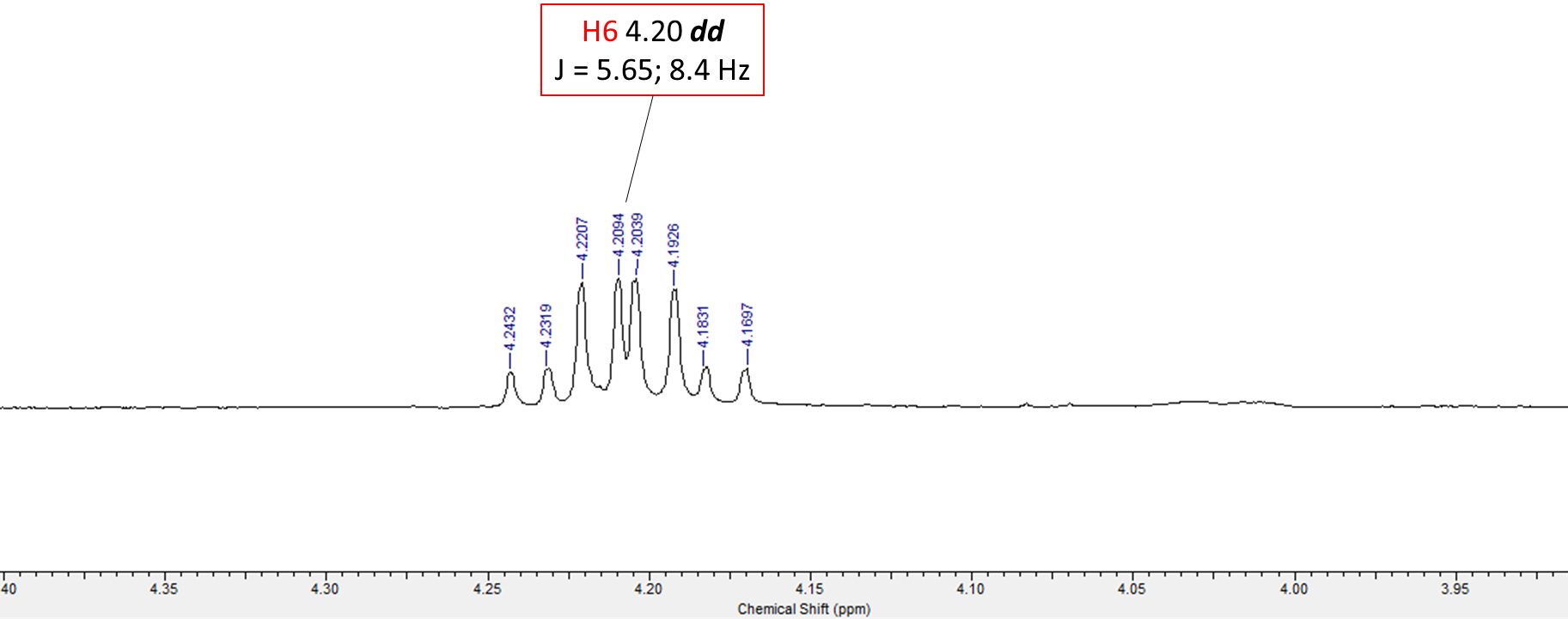


**^1^H NMR sample 070 Expansion II (CDCl_3_. 500 MHz)**


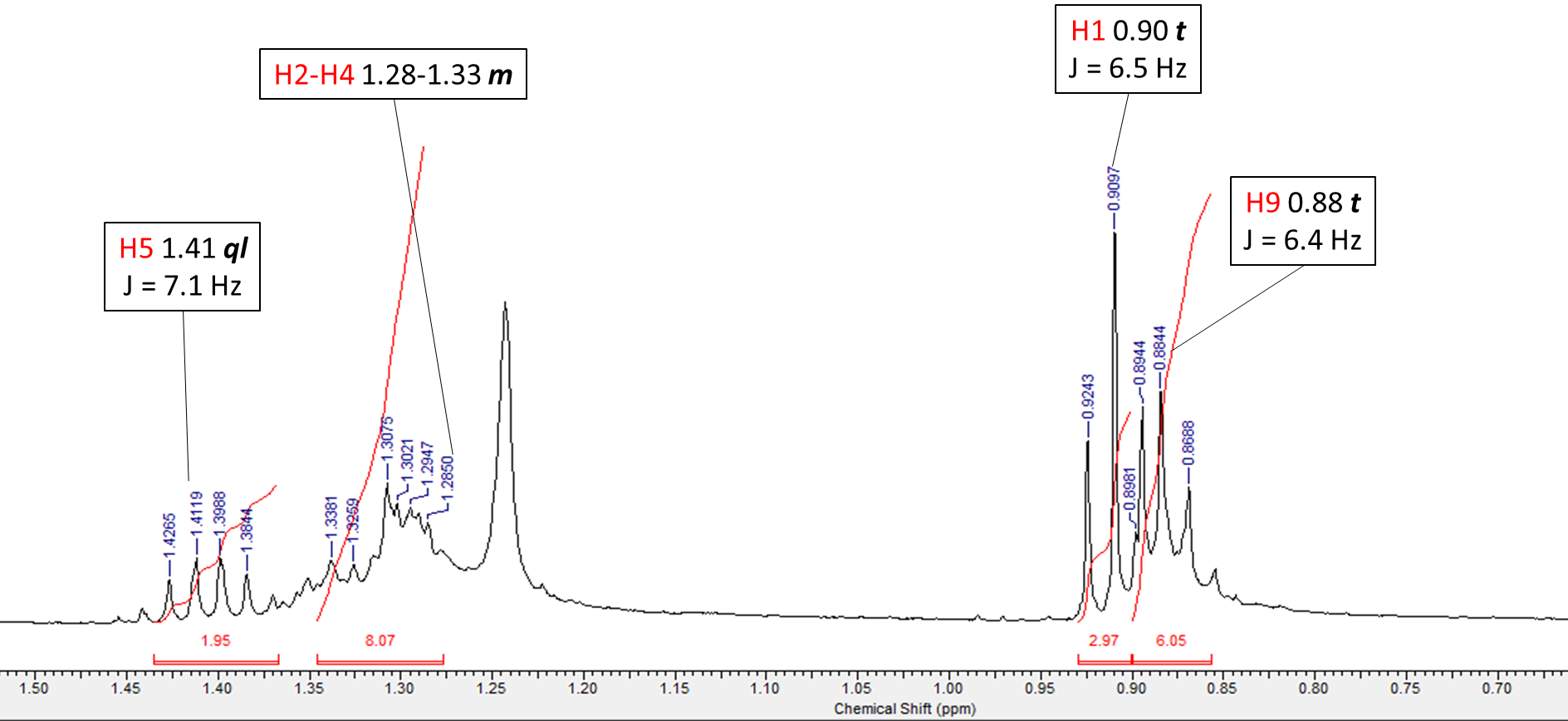


**^1^H NMR sample 070 Expansion III (CDCl_3_. 500 MHz)**


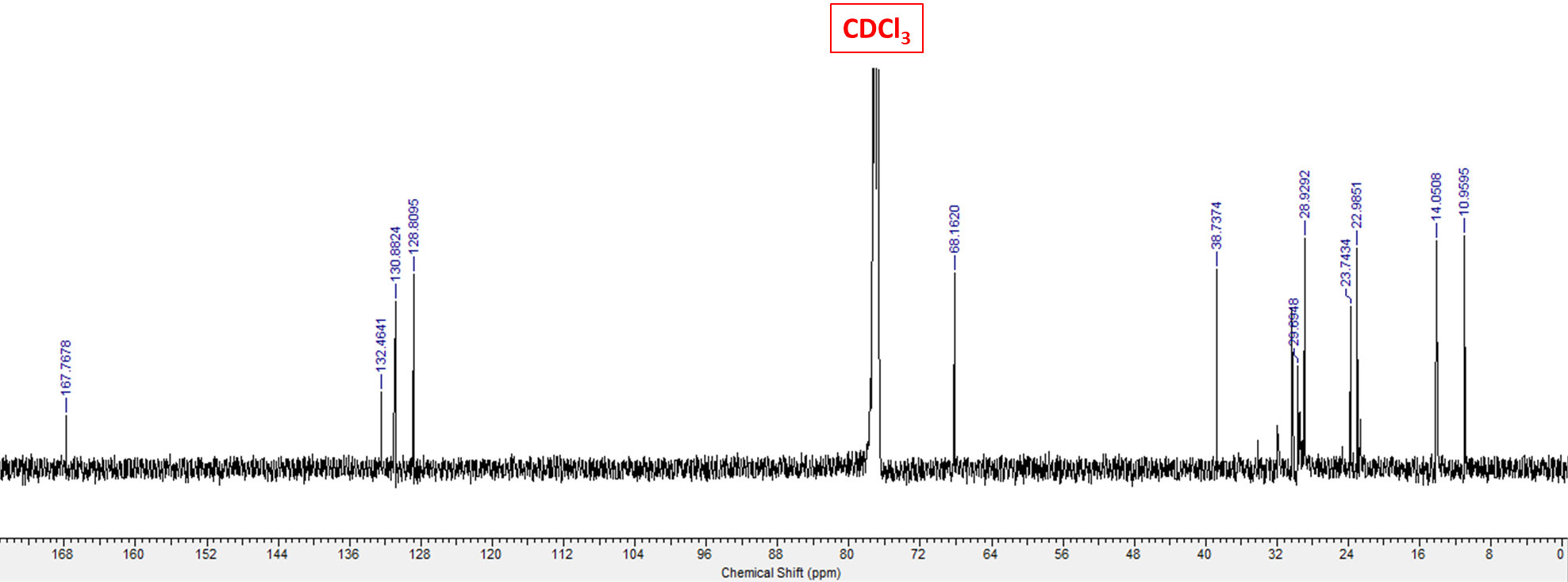


**^13^C NMR sample 070 (CDCl_3_. 125 MHz)**


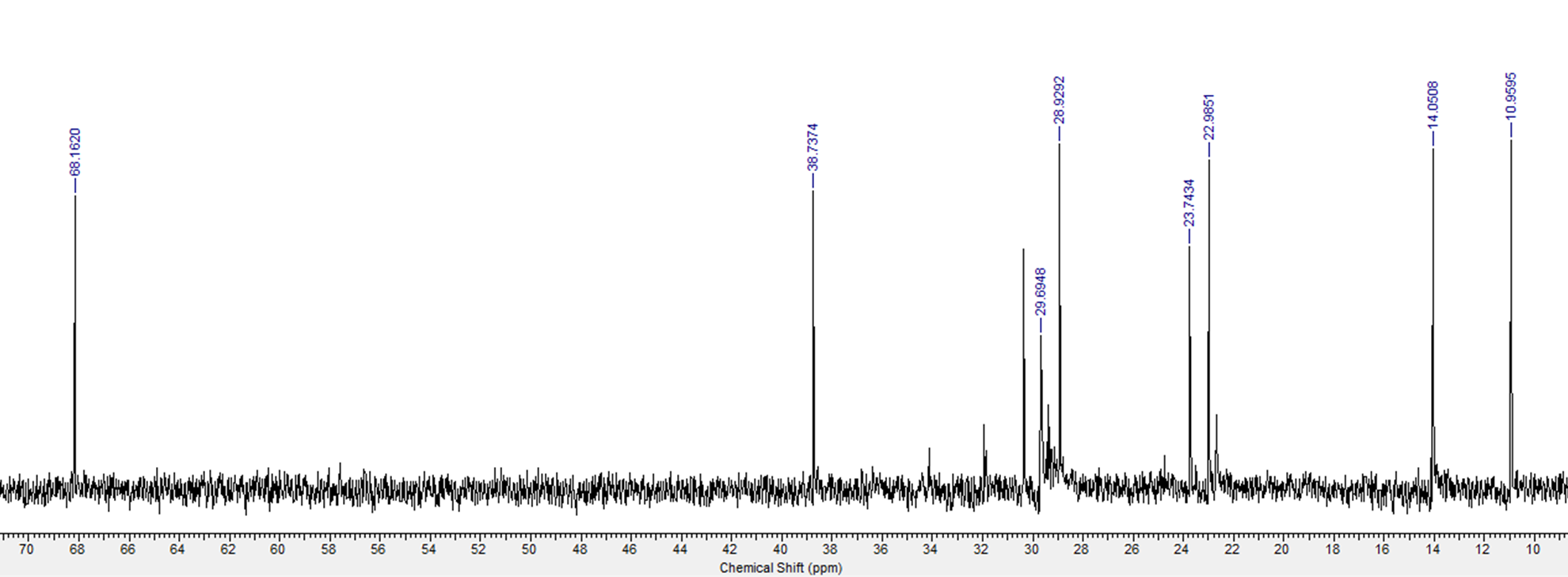


**^13^C NMR sample 070 Expansion I (CDCl_3_. 125 MHz)**


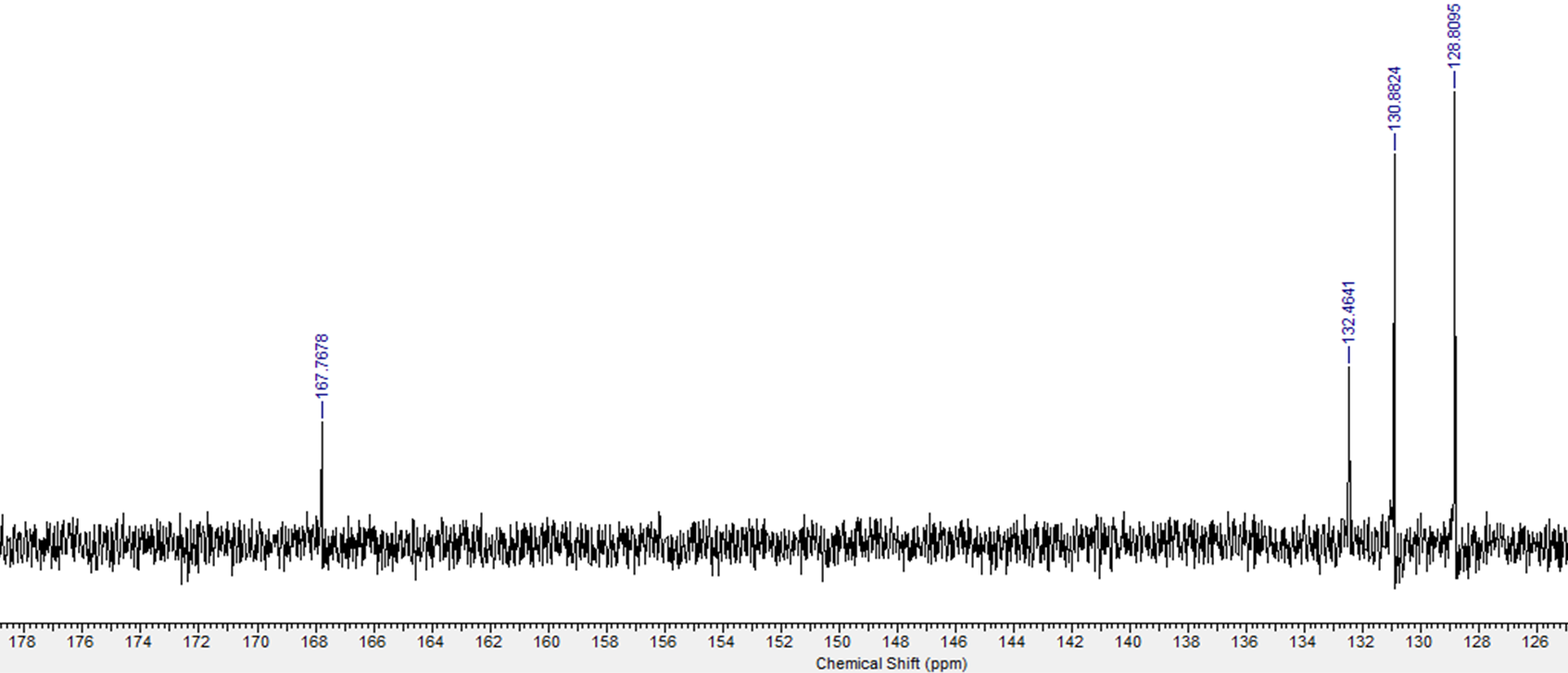


**^13^C NMR sample 070 Expansion II (CDCl_3_. 125 MHz)**


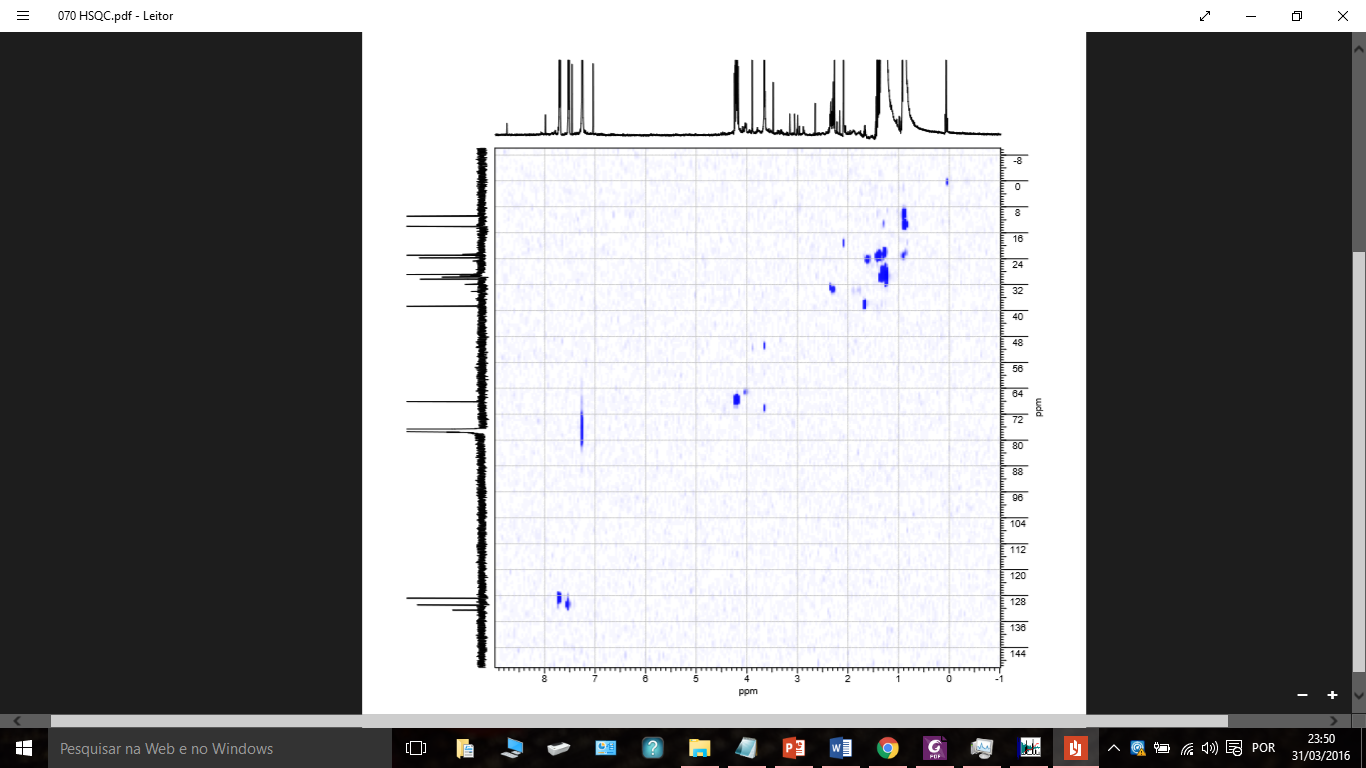

**HSQC sample 070 (500 MHz - ^1^H. 125 MHz - ^13^C. CDCl_3_)**


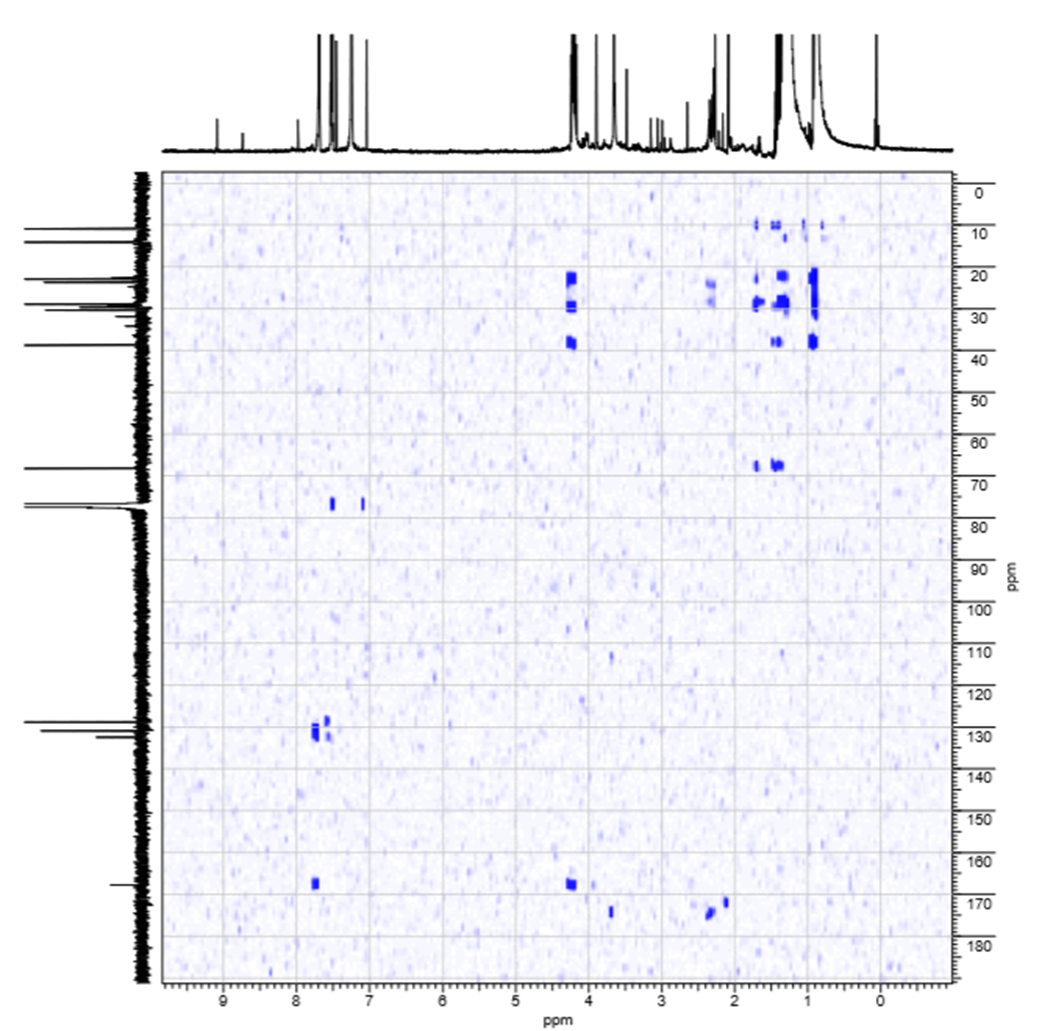

**HMBC sample 070 (500 MHz - ^1^H. 125 MHz - ^13^C. CDCl_3_)**

**
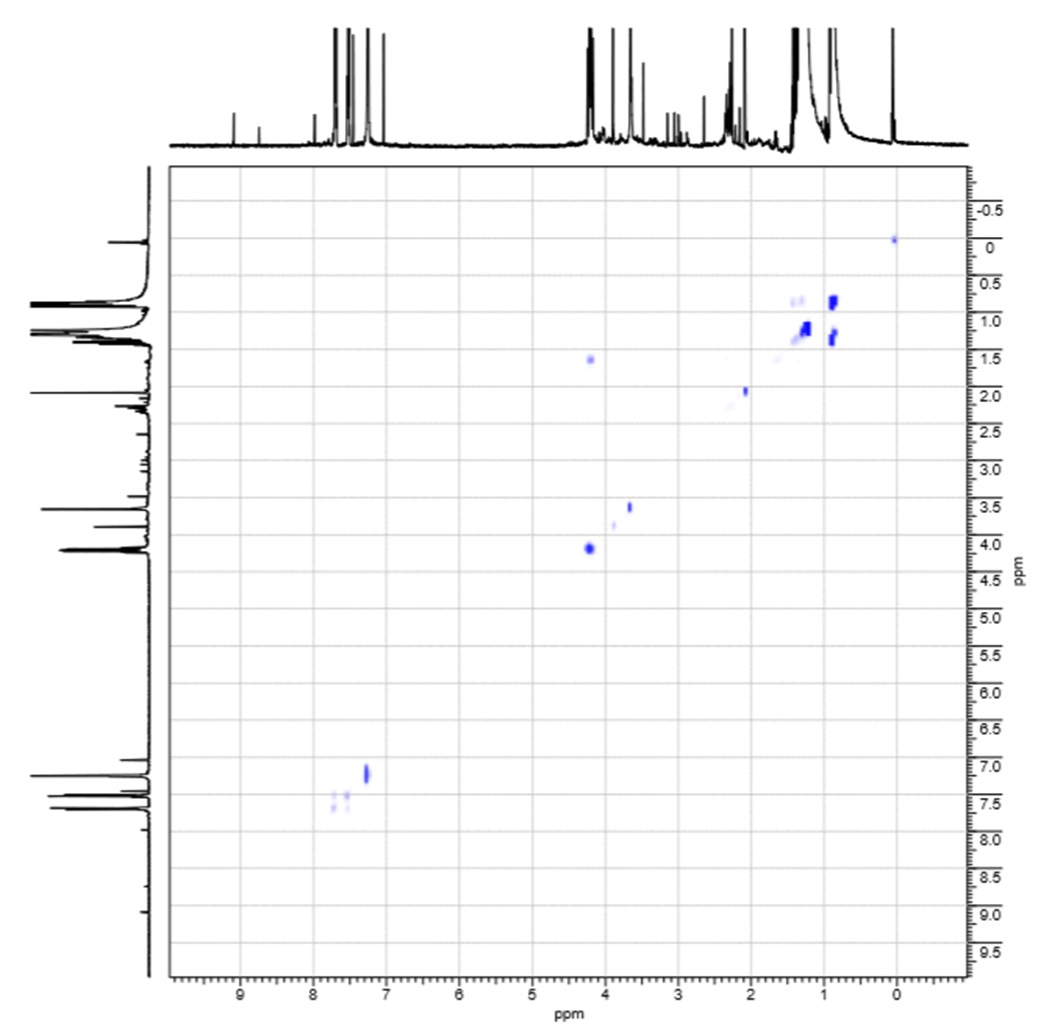

COSY sample 070 (500 MHz. CDCl_3_)**
