# Supplementary figures and images for: Diversity of cultivable fungal endophytes in Paullinia cupana (Mart.) Ducke and bioactivity of their secondary metabolites
Source: PLoS One. 2018 Apr 12;13(4):e0195874. doi: 10.1371/journal.pone.0195874 (PMC5897019; doi:10.1371/journal.pone.0195874)

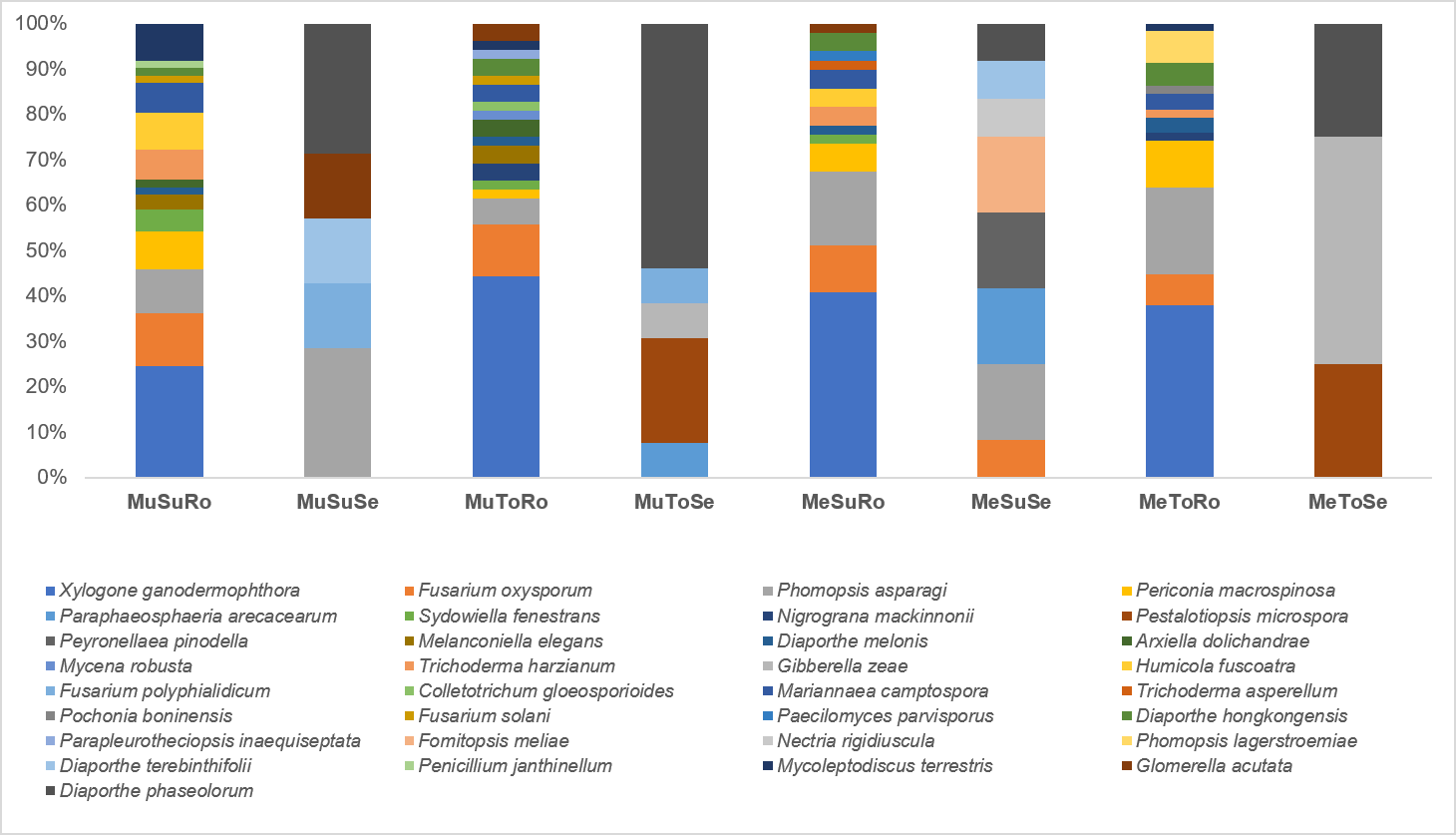

Supplement: S1 Fig — Mu = Manaus; Me = Maués; Su = Susceptible phenotype (CMU 300); To = Tolerant phenotype (CMU 871); Se = Seeds; Ro = Roots. (TIF) [file pone.0195874.s003.tif]

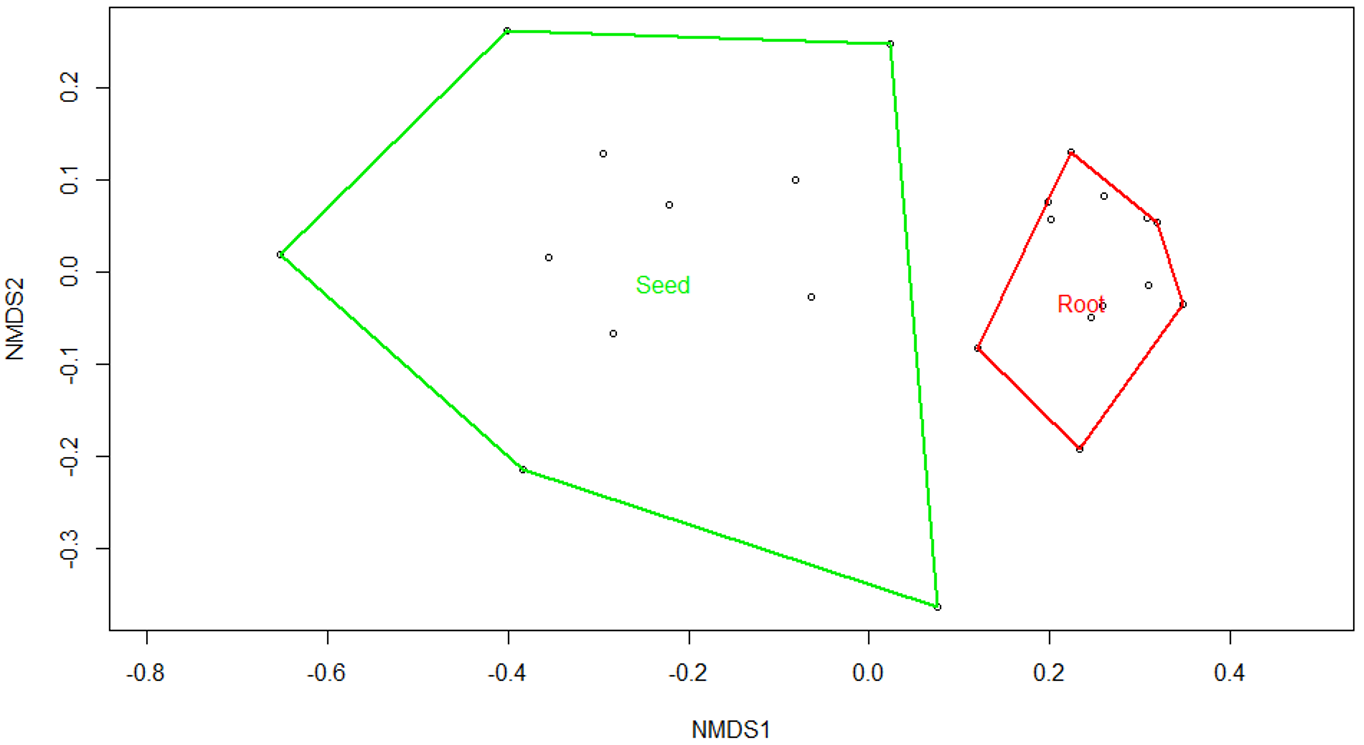

Supplement: S2 Fig — Each point represents a single endophytic community. Permutation tests resulted a highly significant classification (P = 0.001). The lines separate communities from seeds and roots. (TIF) [file pone.0195874.s004.tif]

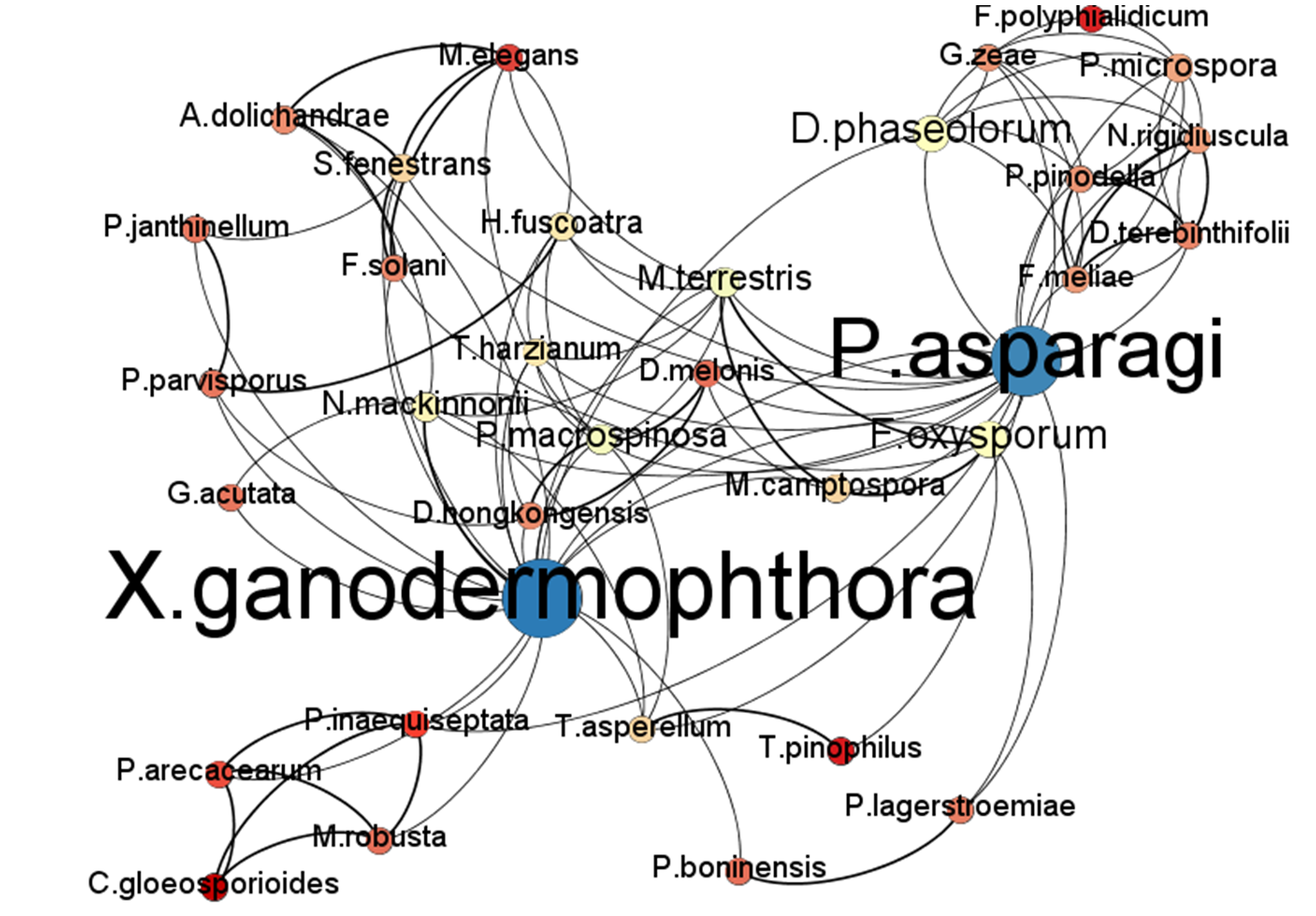

Supplement: S3 Fig — The size of each node is proportional to its betweeness centrality. Blue, yellow, and red nodes indicate a high, intermediate, and low degree of betweenness centrality, respectively. Thick lines represent positive (Spearman’s ρ>0.6) and significant (P<0.05) correlations. Thin lines represent positive non-significant correlations (P>0.05). (TIF) [file pone.0195874.s005.tif]

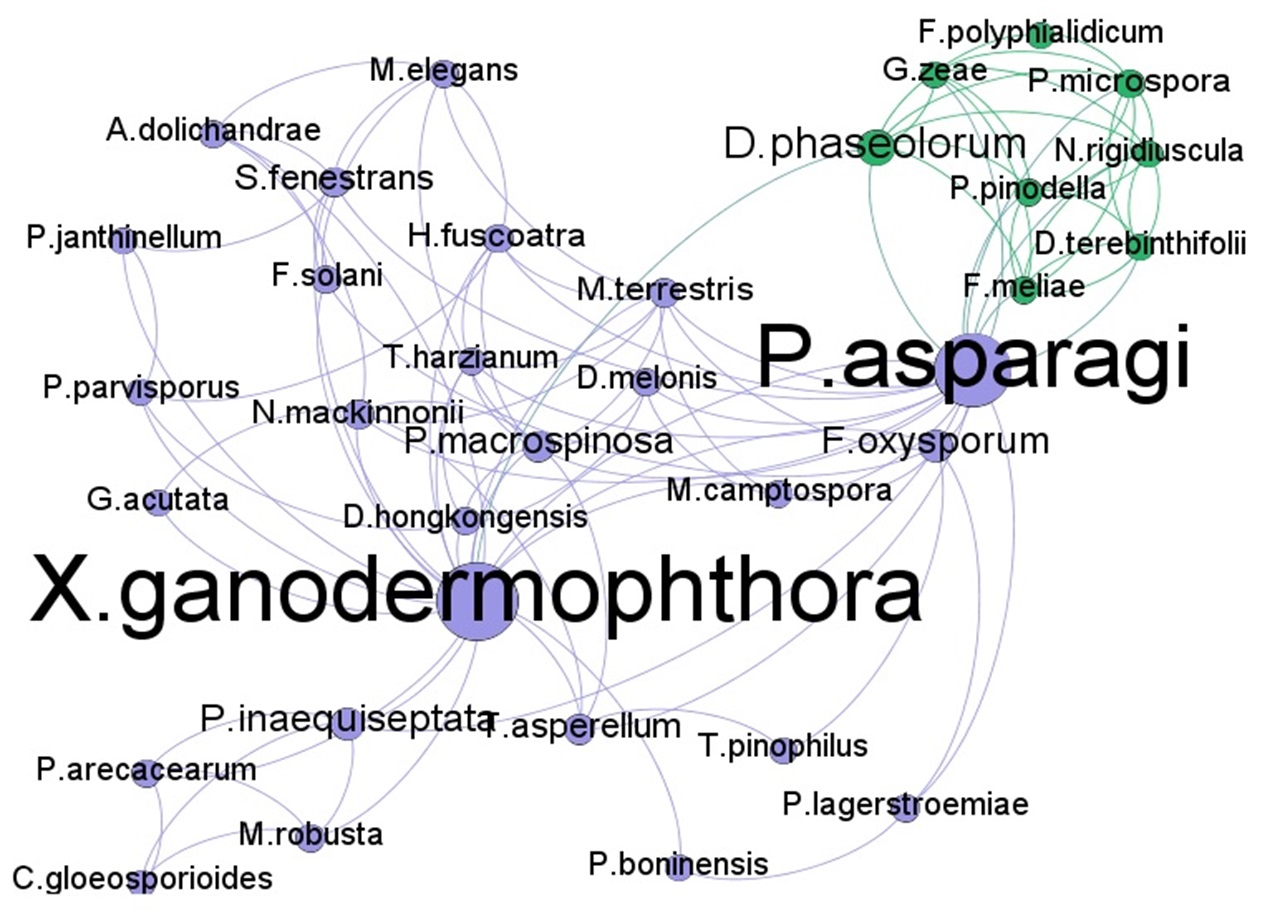

Supplement: S4 Fig — Different colors represent the two modular communities identified. Green and blue modules represent the species isolated from seeds and roots, respectively (modularity index = 0.303, P < 0.01). (TIF) [file pone.0195874.s006.tif]

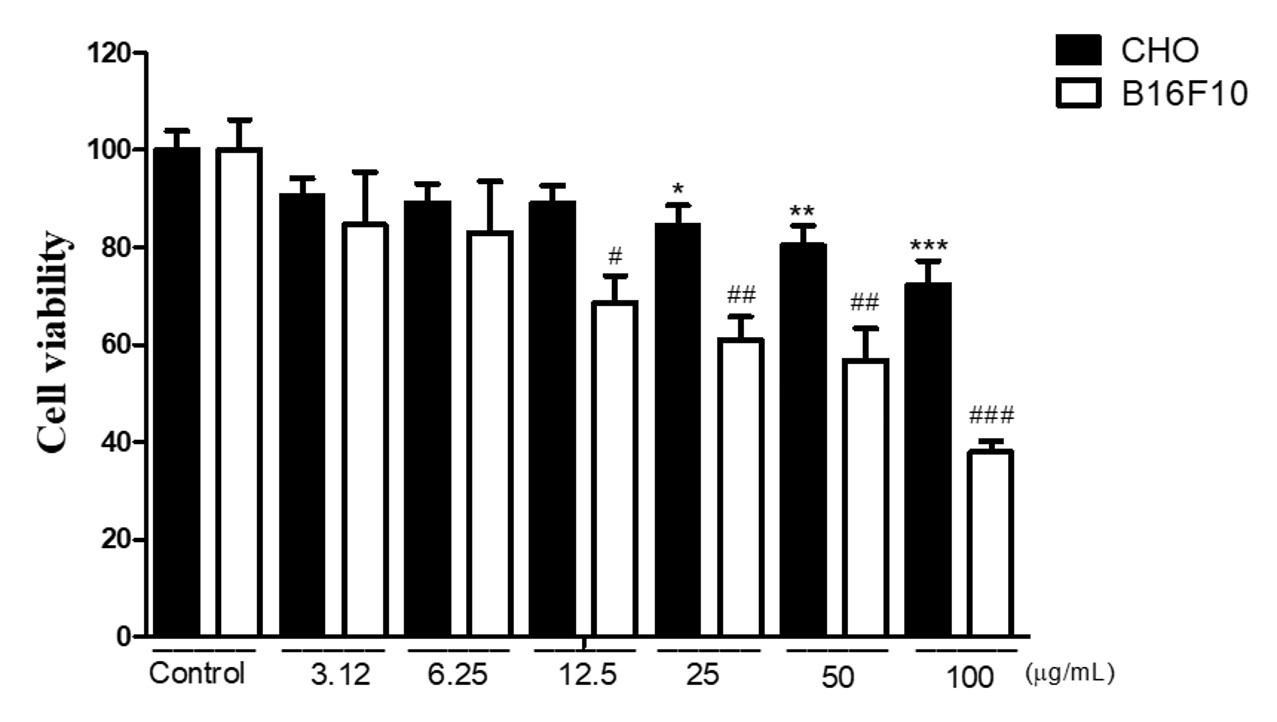

Supplement: S5 Fig — *(CHO) and #(B16F10): P < 0.05 vs. control (untreated cells); Dunnett's multiple comparison test. (TIF) [file pone.0195874.s007.tif]

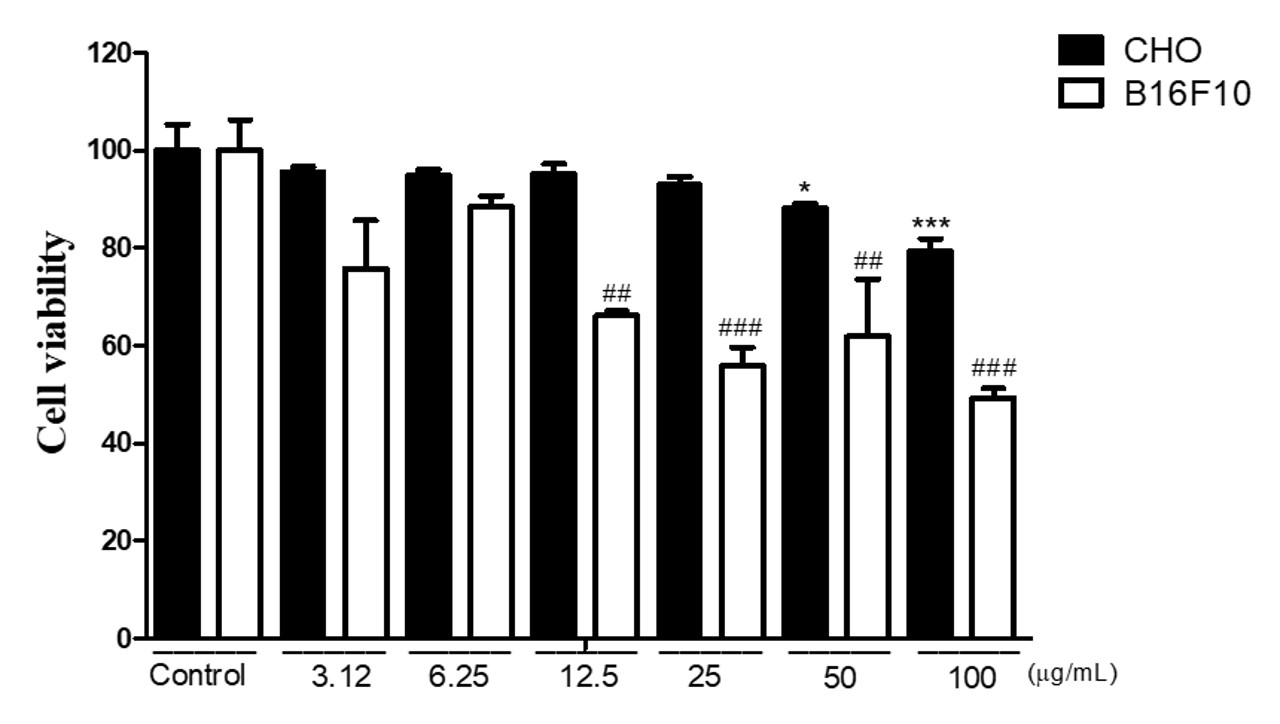

Supplement: S6 Fig — *(CHO) and #(B16F10): P < 0.05 vs. control (untreated cells); Dunnett's multiple comparison test. (TIF) [file pone.0195874.s008.tif]

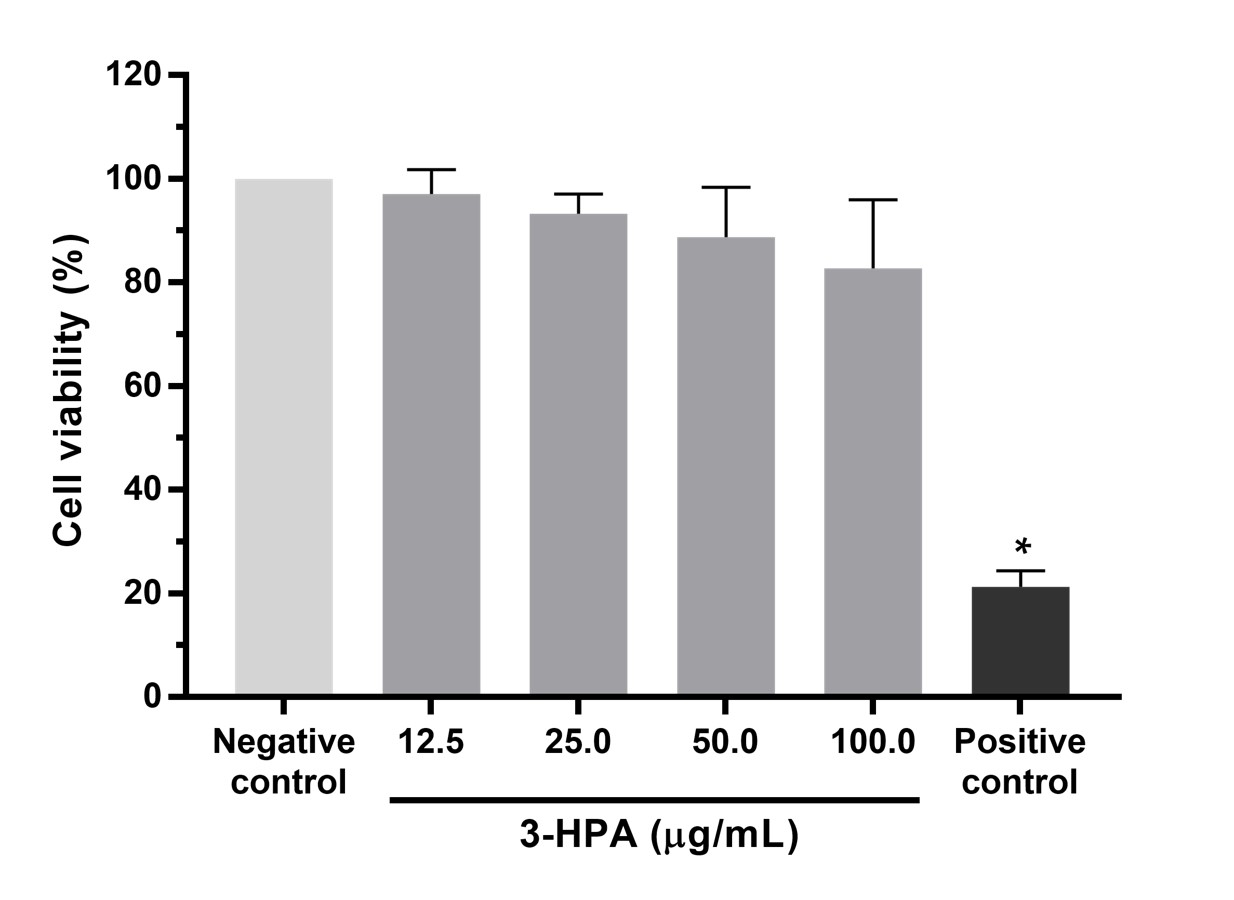

Supplement: S7 Fig — Negative control: untreated cells. Positive control: 10 μM doxorubicin. *P < 0.05 vs. negative control (Dunnett's multiple comparison test). (TIF) [file pone.0195874.s009.tif]

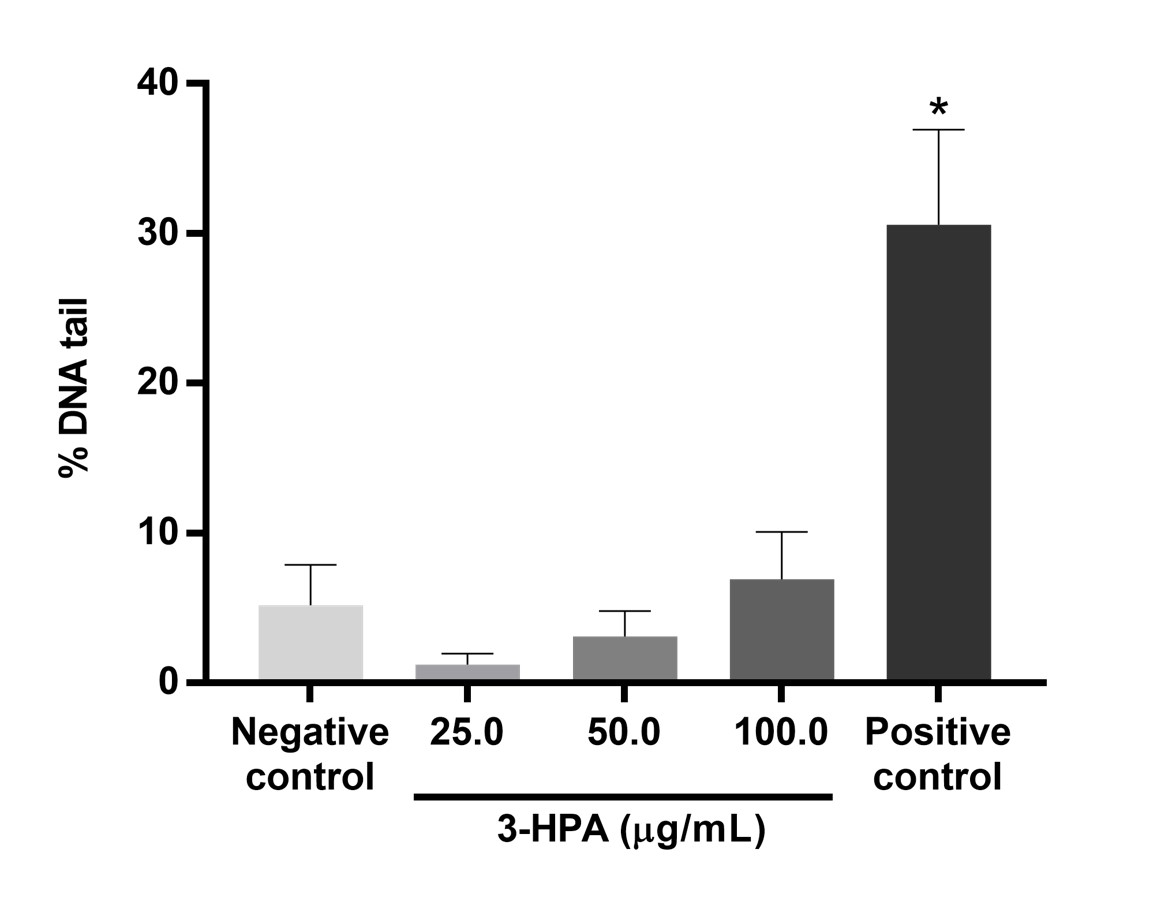

Supplement: S8 Fig — Negative control: untreated cells. Positive control: 200 μM hydrogen peroxide. * P < 0.05 vs. negative control (Dunnett's multiple comparison test). (TIF) [file pone.0195874.s010.tif]
